# Selection on sperm size in response to promiscuity and variation in female sperm storage organs

**DOI:** 10.1111/jeb.14120

**Published:** 2022-11-10

**Authors:** Emily R. A. Cramer, Zelealem B. Yilma, Jan T. Lifjeld

**Affiliations:** ^1^ Sex and Evolution Research Group, Natural History Museum University of Oslo Oslo Norway; ^2^ Carnegie Mellon University in Qatar Doha Qatar

**Keywords:** cryptic female choice, mate choice, sperm length, sperm morphology, sperm storage

## Abstract

Sperm cells are exceptionally morphologically diverse across taxa. However, morphology can be quite uniform within species, particularly for species where females copulate with many males per reproductive bout. Strong sexual selection in these promiscuous species is widely hypothesized to reduce intraspecific sperm variation. Conversely, we hypothesize that intraspecific sperm size variation may be maintained by high among‐female variation in the size of sperm storage organs, assuming that paternity success improves when sperm are compatible in size with the sperm storage organ. We use individual‐based simulations and an analytical model to evaluate how selection on sperm size depends on promiscuity level and variation in sperm storage organ size (hereafter, female preference variation). Simulations of high promiscuity (10 mates per female) showed stabilizing selection on sperm when female preference variation was low, and disruptive selection when female preference variation was high, consistent with the analytical model results. With low promiscuity (2–3 mates per female), selection on sperm was stabilizing for all levels of female preference variation in the simulations, contrasting with the analytical model. Promiscuity level, or mate sampling, thus has a strong impact on the selection resulting from female preferences. Furthermore, when promiscuity is low, disruptive selection on male traits will occur under much more limited circumstances (i.e. only with higher among‐female variation) than many previous models suggest. Variation in female sperm storage organs likely has strong implications for intraspecific sperm variation in highly promiscuous species, but likely does not explain differences in intraspecific sperm variation for less promiscuous taxa.

## Graphical abstract

We modelled populations where among‐female variation in sperm storage organ (SSO) size (red) was relatively high or low, compared to among‐male variation in sperm size (blue). The form of selection on sperm depended on the interaction between among‐female SSO variation and female promiscuity level (i.e. number of sperm competitors).

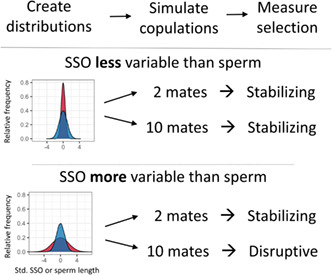

## 1 INTRODUCTION

Sperm cells have exceptional morphological diversity across species (Pitnick et al., 2009). This diversity is partly driven by fertilization environment (internal vs. external; Kahrl, Snook, et al., 2021) and is also hypothesized to be driven by post‐copulatory sexual selection, which can arise when a female copulates with multiple males in a single reproductive bout (Eberhard, 1996; Lupold et al., 2016; Parker, 1970). With such female promiscuity, sperm from different males may compete to fertilize the egg(s) (Parker, 1970) and/or the female may exert cryptic choice for particular sperm or male characteristics (Eberhard, 1996). How such post‐copulatory sexual selection processes result in selection on sperm morphology requires more study in most study systems (Lüpold & Pitnick, 2018), but two patterns are quite robust across studies. Specifically, sperm cell morphology co‐evolves with the morphology of female sperm storage organs both in comparative studies (Dybas & Dybas, 1981; Briskie & Montgomerie, 1992; Higginson et al., 2012; reviewed in Lüpold & Pitnick, 2018) and in experimental evolution studies (e.g. Hosken et al., 2001; Miller & Pitnick, 2002). These studies suggest that sperm evolve to ‘fit’ sperm storage organs (and/or vice versa) in internally fertilizing species (and/or may be positively genetically correlated, e.g. Lupold et al., 2016). In addition, among‐male variation in sperm length is lower in more promiscuous taxa, suggesting stronger selection for an optimal sperm phenotype (sperm total length: birds, Calhim et al., 2007; Lifjeld et al., 2010; rodents, Varea‐Sánchez et al., 2014; and social insects, Fitzpatrick & Baer, 2011; flagellum length: sharks, Rowley, Locatello, et al., 2019). In this paper, we use simulations and an analytical model to explore how promiscuity level and among‐female variability in the sperm storage organs interact in driving selection on sperm.

Female sperm storage organs represent an important selective environment for sperm cells in many species. Correlations between individual males' proportion of sperm stored and proportion of eggs fertilized can be high, reinforcing the idea that successful interaction with the female is important (Bretman et al., 2009; Manier et al., 2010; Hemmings & Birkhead, 2017; though note that females do not necessarily use stored sperm from all males, for exmaple Simmons & Beveridge, 2010; Turnell & Shaw, 2015). Many factors may impact the successful storage of sperm, including motility as the sperm enter the sperm storage organ (Mendonca et al., 2019), mating order (Hellriegel & Bernasconi, 2000; Hemmings & Birkhead, 2017; Manier et al., 2010), complex biochemical interactions among ejaculates and with the female (den Boer et al., 2010), and genetic compatibility of the male and female (Simmons et al., 2006; Gasparini & Pilastro, 2011; though genetic compatibility may be assessed in the male rather than directly from the sperm, Løvlie et al., 2013). Here we focus on the potential impact of morphological compatibility between the sperm cell and the sperm storage organ, which is suggested by the coevolution of morphology of sperm and sperm storage organs across taxa (reviewed in Lüpold & Pitnick, 2018). There are notable exceptions to the idea of morphological compatibility; for example, García‐González and Simmons (2007) find stronger selection for short sperm in females with larger sperm storage organs in the dung beetle *Onthophagus taurus*, and honey bee *Apis mellifera* females have two large sac‐like spermathecae that each can store millions of sperm (Snodgrass, 1910), where close physical associations between the female sperm storage organ and individual sperm cells seems unlikely. The mechanism we outline here will, therefore, not be applicable in all systems, but appears reasonable in birds and other species where sperm orient parallel to the female sperm storage organs, with a relatively modest number of sperm per storage compartment.

In addition to being important selective environments for sperm, female sperm storage organs likely vary among individuals, following several lines of evidence. First, since genetic variation is a pre‐requisite for evolution, the fact that sperm storage organ morphology evolves suggests that it varies (Jennions & Petrie, 1997). Genetic variation in sperm storage organ morphology has also been directly documented (Lüpold et al., 2013; Miller et al., 2003; Miller & Pitnick, 2002). In addition, environmental and social conditions during development can affect sperm storage organ morphology (Amitin & Pitnick, 2007; Berger et al., 2011; Farrow et al., 2022). Within‐female variation is also known, for example, in birds, where each female has hundreds of sperm storage tubules, whose lengths vary in a gradient across the utero‐vaginal junction (where these structures occur) and with stage of the egg‐laying cycle (Briskie, 1996).

Thus we hypothesize that females vary in their sperm storage organ morphology and that the morphological fit between these organs and sperm cells is a mechanism of cryptic female choice, because it biases storage success (and, therefore, fertilization success) towards well‐fitted sperm. We model a scenario where all females have the same preference function, whereby the sperm that best fit their sperm storage organs is more likely to fertilize their eggs. However, females' preferred male phenotypes (i.e. the sperm size that best fit individual females) vary because the preference function is self‐referential against a variable morphological trait. This hypothesis is supported by Hemmings et al. (2016), who allowed females to copulate with one male and then compared the morphology of ejaculated cells and of sperm cells that reached the ovum after sperm storage. Re‐analysis of their data (Hemmings et al., 2018, see Appendix S1) indicates that the mean sperm length at the egg differed from the mean ejaculated sperm in 9 of 27 females (Figure S1). Sperm at the egg were longer than ejaculated sperm for approximately half the females and shorter in the other half, consistent with variable female preferences for sperm size. Furthermore, under this hypothesis, we can expect that males may have different relative fertilization success when they copulate with different females. Several studies do indeed find that the combination of male and female identities (or genetic lines) has a strong impact on fertilization success (Birkhead et al., 2004; Bjork et al., 2007; Clark, 2002; Reinhart et al., 2015; Simmons et al., 2014; Wilson et al., 1997) (although we note that a combinatorial effect of male and female may also arise due to diverse other processes, for example, variation in copulation duration, Eady & Brown, 2017; sperm swimming speed, Urbach et al., 2005; Cramer et al., 2014, 2016; and sperm ejection by the female Lüpold et al., 2020).

Because we view the fit between sperm and sperm storage organ as a mechanism of cryptic female choice (Lüpold & Pitnick, 2018), we can expect some parallels between this process and mate choice. However, to our knowledge, no theoretical work on mate choice models can be directly applied to cryptic female choice, because none use the conditions most relevant for sperm‐female interactions. Specifically, most mate choice models assume that females copulate with a single male in the population, while empirical data show that females often copulate with multiple males, who then share paternity of their offspring (e.g. Brouwer & Griffith, 2019; Gage, 1994; Kahrl, Kustra, et al., 2021; Simmons et al., 2007; Simmons & Beveridge, 2010; Turnell & Shaw, 2015). In addition, we assume that females copulate with fewer males than they assess during mate choice, implying that females sample the sperm of relatively few males. The number of sampled partners is known to impact resulting selection strength (Gomulkiewicz, 1991; Janetos, 1980; Muniz & Machado, 2018). Finally, in species where eggs are ovulated in batches, female sperm storage organs have already gathered all the sperm cells that potentially can fertilize the eggs, making cryptic female choice best represented by a simultaneous assessment model. Under a simultaneous assessment strategy, the female evaluates all individuals in a set of potential males before choosing among them. Simultaneous assessment strategies can give different results from other assessment strategies (Janetos, 1980; Jennions & Petrie, 1997; Muniz & Machado, 2018), and to the best of our knowledge, continuous variation in female preferences has not been modelled with simultaneous assessment with a reasonable (for an internally fertilizing species) number of copulation partners; see Millan et al. (2020) for relevant work with a different assessment model, and Van Doorn et al. (2001) and van Doorn and Weissing (2002) for models relevant for broadcast spawners with high mate sampling. Further work is thus needed to understand how variation in female sperm storage organs impacts selection on sperm.

Here, we use individual‐based simulations and an analytical model to investigate how among‐female variation in sperm storage organs affects the resulting selective pressure on sperm, and we assess whether this relationship depends on the level of female promiscuity, that is, the number of copulation partners. We predict that selection will be stronger with higher promiscuity (Gomulkiewicz, 1991; Janetos, 1980; Muniz & Machado, 2018). We further hypothesize that where female sperm storage organs are less variable than sperm, there will be stronger stabilizing selection on sperm as female trait variation is further reduced. Conversely, where female sperm storage organs are more variable than sperm, we predict that there will be stronger disruptive selection on sperm as variation in the female trait increases (Jennions & Petrie, 2000; Van Doorn et al., 2001; van Doorn & Weissing, 2002; Weissing et al., 2011). Our model is formulated around the mechanism of sperm storage in passerine songbirds, but the model could be applied to other taxa and contexts where the match between a male phenotype and a female template is important for male reproductive success.

## 2 METHODS

### 2.1 Assumptions

We assume a closed population with an equal sex ratio, where copulations occur randomly with respect to the sperm and sperm storage organ sizes. All eggs are fertilized, so that preference is selectively neutral for females. This assumption is similar to the ‘last‐chance’ option of Janetos (1980), whereby females accept any male rather than not mate.

### 2.2 Simulation procedure

**FIGURE 1:**
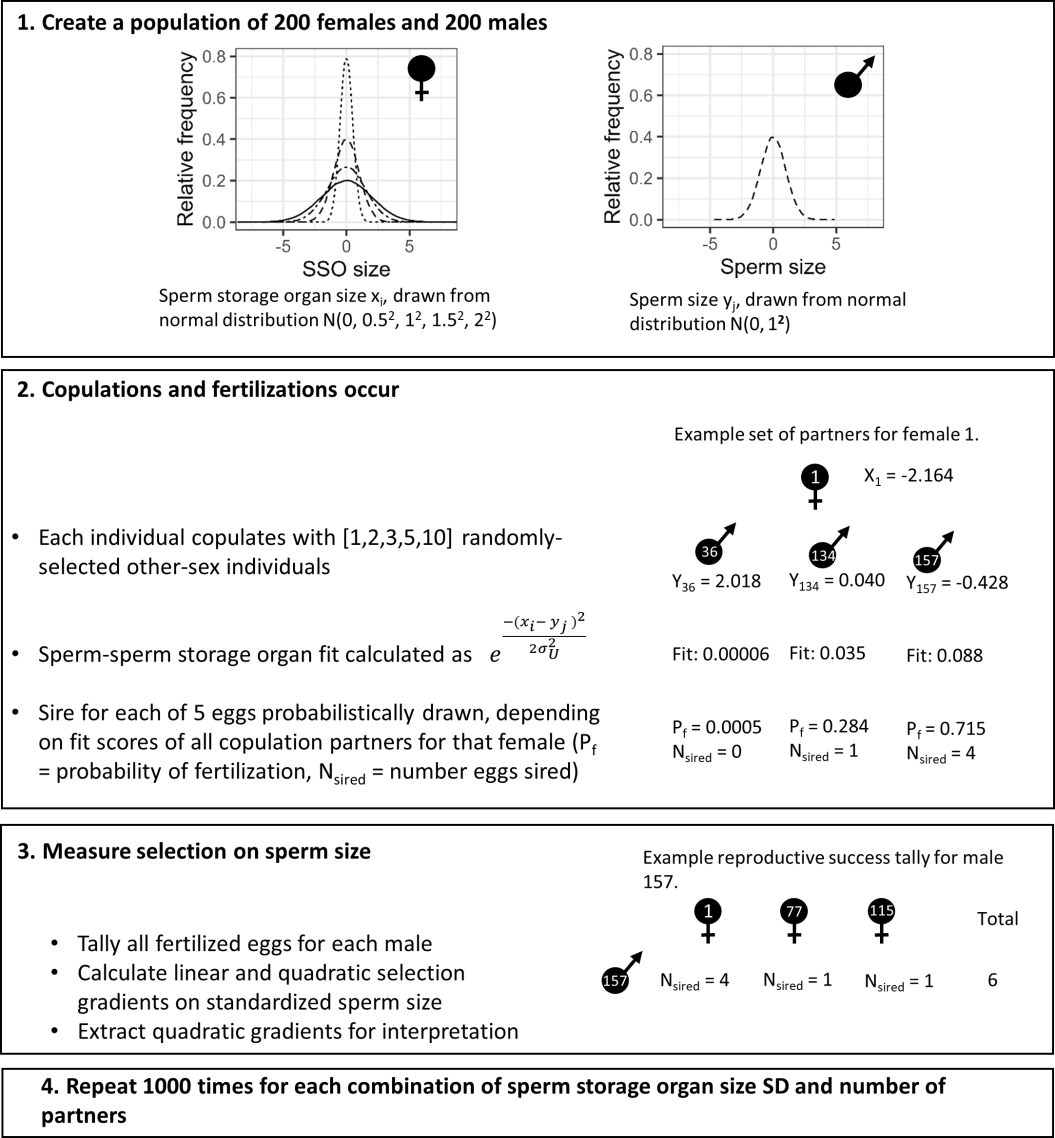
Diagram of simulation steps, with values in square brackets for variables that had different values in different populations in the main simulations. The value of $\sigma_{U}^{2}$ was 1 for the main simulations. For steps 2 and 3, an example for a focal female and male (respectively) are shown. Note that size is given relative to the population mean of 0.

For each iteration of the simulation, we created a population of 200 individuals of each sex, breeding for one season (Step 1, Figure 1). Each female produced one set of 5 eggs. Males were assigned a sperm size from a normal distribution with mean = 0 and SD = 1. Females were assigned a sperm storage organ size on the same scale, such that the fit between sperm and sperm storage organ was best when the trait values were equal. We varied population‐level SD in sperm storage organ (values of 0.5, 1, 1.5 and 2; comparable to the variation explored by Millan et al., 2020), but, for simplicity, the population mean sperm storage organ size was always equal to the mean sperm size.

All individuals copulated with 1, 2, 3, 5, or 10 different members of the opposite sex (Figure 1, Step 2). Detailed information on number of copulation partners is poorly known for many species and is often inferred from genotyping stored sperm in the female or determining paternity of offspring. Empirical data thus provides a minimum estimate of number of individual partners (Cramer, Kaiser, et al., 2020). For many species, an average number of copulation partners less than 5 appears realistic (Brommer et al., 2007, 2010; Cramer, Kaiser, et al., 2020; Gage, 1994; Kahrl, Kustra, et al., 2021; Rowley, Daly‐Engel, et al., 2019; Simmons et al., 2007; Simmons & Beveridge, 2010; Turnell & Shaw, 2015). The values we chose to investigate were also informed by the expectation that selection strength should asymptote with > about 10 copulation partners (Gomulkiewicz, 1991; Muniz & Machado, 2018). We include 1 copulation partner to confirm the expectation of no selection on sperm under this condition. Copulation partners were assigned randomly by shuffling the list of individual identities for each copulation event. After each shuffle, we checked whether any male appeared as a copulation partner twice for any female, and if so, we re‐shuffled all identities for that copulation event. Thus, for each value of *n* copulations, each female copulated with *n* different males, and each male copulated with *n* different females.

Following copulation, the fertilizing sperm for each egg was determined following a loaded raffle (sensu Parker, 1990), where loading is by the relative fit of the sperm‐female sperm storage organ (Figure 1, Step 2). All ejaculates were assumed to contain equal numbers of sperm, and each male–female pair copulated only once, so that sperm number did not vary among competing males. We assume no precedence related to copulation order. To create the loaded raffle, we used R's *sample* function, which requires positive, non‐zero values as weights; it then sums all individuals' weight values, and the probability that an individual is drawn is proportional to its contribution to the sum of the weight values across all individuals. Thus, a male's success depends on his relative fit for the female sperm storage organ compared to the other copulation partners, not his absolute fit. To calculate absolute fit, we modelled fit as a Gaussian function, which attains its maximum when the male's sperm size, *y*, matches the female's sperm storage organ, *x*. The parameter $\sigma_{U}$, akin to standard deviation, controls the strength of the preference for well‐fitting sperm (sensu Millan et al., 2020). For simplicity, we use a value of $\sigma_{U}$ = 1 in all simulations. We, therefore, calculated the fit score between the sperm size, *y*, and the female sperm storage organ, *x*, as:

(1) $$e^{\frac{-{\left(x-y\right)}^{2}}{{2\sigma }_{U}^{2}}}$$

This equation represents the preference function used by all females. After calculating the fit for all copulation partners, we assigned fertilization by drawing male identities from a list of the individual female's copulation partners, weighted according to the fit scores (written out in Figure 1, Step 2, but accomplished via the *sample* function in R).

After counting all offspring sired for each male, the selection gradient on sperm size was calculated (Figure 1, Step 3). To do so, reproductive success was standardized by dividing by the population mean reproductive success. Sperm size was standardized to have a population mean of 0 and standard deviation of 1 (following Lande & Arnold, 1983). Standardized reproductive success was then regressed on standardized sperm size, including both a linear and a quadratic term (Lande & Arnold, 1983). Negative values of the quadratic term indicate stabilizing selection, and positive values indicate disruptive selection. We extracted the quadratic selection gradient parameter from each replicate population.

After performing 1000 replicate populations with the same set of conditions, we compared how the quadratic selection gradient changed with the treatments (variation in female sperm storage organ and number of copulation partners). To facilitate interpretation, we treat each predictor (i.e. variation in the female sperm storage organ, number of copulation partners and their interaction) as categorical rather than continuous. The estimated quadratic selection coefficient from each population was the response variable. We used linear models with no random effects, since each simulated population should be independent, and sample sizes were identical across treatments. This approach implicitly treats each simulated population as equally distantly related to all other simulated populations (i.e. different populations in one species, or different species with a star‐shaped phylogeny). Following the logic outlined in White et al. (2014), we rely on effect size estimates rather than *p*‐values in interpreting our results (since simulations can make sample size be arbitrarily high and *p*‐values correspondingly low). Following Richardson (2011), we use *η* ^2^ as the effect size estimate, with values of 0.1, 0.25 and 0.5 considered small, medium and large, respectively. These were calculated via sjstats (Lüdecke, 2021). We further directly calculated the 95% quantile limits (i.e. 0.5% and 97.5% quantiles of the observed values) for each simulation condition.

All simulations and statistics were performed in R (v 4.1.1) (R Development Core Team, 2020) and RStudio (2022.02.3+492) using base functions and the tidyverse package (Wickham et al., 2019). In addition, we ran a modified set of simulations to assess the impact of (1) a social‐pair partner that obtains repeated copulations with a female; (2) a threshold effect, whereby some sperm fit the sperm storage organs too poorly to be stored and thus do not fertilize eggs; (3) directional selection for larger sperm, combined with selection for well‐fitting sperm; (4) variation in $\sigma_{U}$, as a proxy for the effect of within‐female variation in sperm storage organ size; (5) allowing one male to sire all offspring; and (6) separately altering male and female promiscuity levels. Overall patterns were highly similar (Figures S2–S14). Code necessary to replicate our main results (and most supplementary conditions) is archived on Zenodo (https://doi.org/10.5281/zenodo.7128886).

### 2.3 Analytical model

Among‐female variation in female sperm storage organs had strong impacts on the shape of selection (see Section 3), which depended also on the number of copulation partners. To better understand when disruptive or stabilizing selection should be expected when the female could sample all males, we used an analytical model that parallels the simulation. Similar to the simulations, among‐female variation in sperm storage organs is modelled as normally distributed with mean of 0 and standard deviation $\sigma_{F}$. Among‐male variation in sperm is modelled as normally distributed with mean 0 and standard deviation $\sigma_{M}$. The probability density function of the female sperm storage organ size across all females is then

(2) $$F\left(x\right)=\frac{1}{\sqrt{2\pi }\sigma_{F}}e^{-x^{2}/2\sigma_{F}^{2}}$$

and the probability density function for sperm size across all males is

(3) $$M\left(y\right)=\frac{1}{\sqrt{2\pi }\sigma_{M}}e^{-y^{2}/2\sigma_{M}^{2}}$$

Denoting the preference function as *U*(*x*, *y*), the probability distribution function of fertilization success for all males with sperm size *y*, given a female with storage organ size *x*, can be expressed as

(4) $$S\left(y|X=x\right)=\frac{M\left(y\right)U\left(x,y\right)}{\int_{-\infty }^{\infty }M\left(y\right)U\left(x,y\right)dy}=\frac{M\left(y\right)U\left(x,y\right)}{V\left(x\right)}$$

Intuitively, the denominator, *V*(*x*), can be thought of as the total of the female's fit scores across all males in the population, and the numerator expresses the contribution of males with trait value *y* to the total of the female's fit scores. This is analogous to the *sample* function if all males were sampled.

Fertilization success for all males with trait value *y* can be calculated as the integral of their fertilization success across all females:

(5) $$S\left(y\right)=\int_{-\infty }^{\infty }F\left(x\right)S\left(y|X=x\right)dx=M\left(y\right)\int_{-\infty }^{\infty }\frac{F\left(x\right)U\left(x,y\right)}{V\left(x\right)}dx=M\left(y\right)R\left(y\right)$$

where

(6) $$R\left(y\right)=\int_{-\infty }^{\infty }\frac{F\left(x\right)U\left(x,y\right)}{V\left(x\right)}dx=\frac{S\left(y\right)}{M\left(y\right)}$$

gives the fertilization success of males with value *y*, relative to their representation in the population.

In the simulations, we assumed that *U*(*x*, *y*) was given by Equation (1). Under this condition, we can explicitly calculate the function *R*(*y*). By substituting Equations (1)–(3) into the more general form Equations (4)–(6), we have

(7) $$V\left(x\right)=\frac{1}{\sqrt{2\pi }\sqrt{\sigma_{M}^{2}+\sigma_{U}^{2}}}e^{-\frac{x^{2}}{2\left(\sigma_{M}^{2}+\sigma_{U}^{2}\right)}}$$

(8) $$S\left(y|X=x\right)=\sqrt{\frac{\sigma_{M}^{2}+\sigma_{U}^{2}}{2\pi \sigma_{M}^{2}\sigma_{U}^{2}}}e^{\frac{x^{2}}{2\left(\sigma_{M}^{2}+\sigma_{U}^{2}\right)}-\frac{y^{2}}{2\sigma_{M}^{2}}-\frac{{\left(x-y\right)}^{2}}{2\sigma_{U}^{2}}}$$

(9) $$R\left(y\right)=\frac{\sigma_{M}\sqrt{\frac{1}{\sigma_{M}^{2}}+\frac{1}{\sigma_{U}^{2}}}}{\sigma_{F}\sqrt{\frac{1}{\sigma_{F}^{2}}-\frac{1}{\sigma_{M}^{2}+\sigma_{U}^{2}}+\frac{1}{\sigma_{U}^{2}}}}e^{-\frac{y^{2}\left(-\sigma_{F}^{2}+\sigma_{M}^{2}+\sigma_{U}^{2}\right)}{2\left(\sigma_{F}^{2}\sigma_{M}^{2}+\sigma_{M}^{2}\sigma_{U}^{2}+\sigma_{U}^{4}\right)}}$$

We can rewrite Equation (9) as follows:

(10) $$=\frac{\sigma_{M}^{2}+\sigma_{U}^{2}}{\sqrt{\sigma_{F}^{2}\sigma_{M}^{2}+\sigma_{M}^{2}\sigma_{U}^{2}+\sigma_{U}^{4}}}e^{-\frac{y^{2}\left(-\sigma_{F}^{2}+\sigma_{M}^{2}+\sigma_{U}^{2}\right)}{2\left(\sigma_{F}^{2}\sigma_{M}^{2}+\sigma_{M}^{2}\sigma_{U}^{2}+\sigma_{U}^{4}\right)}}$$

(11) $$=\frac{\sigma_{M}^{2}+\sigma_{U}^{2}}{\sqrt{\sigma_{M}^{2}\left(\sigma_{F}^{2}-\sigma_{M}^{2}-\sigma_{U}^{2}\right)+{\left(\sigma_{M}^{2}+\sigma_{U}^{2}\right)}^{2}}}e^{-\frac{y^{2}\left(-\sigma_{F}^{2}+\sigma_{M}^{2}+\sigma_{U}^{2}\right)}{2\left(\sigma_{F}^{2}\sigma_{M}^{2}+\sigma_{M}^{2}\sigma_{U}^{2}+\sigma_{F}^{4}\right)}}$$

This model is similar to models used by several authors (e.g. Dieckmann & Doebeli, 1999; Lande, 1981), but those authors did not explicitly describe conditions predicting stabilizing and disruptive selection.

## 3 RESULTS

### 3.1 Simulations

**FIGURE 2:**
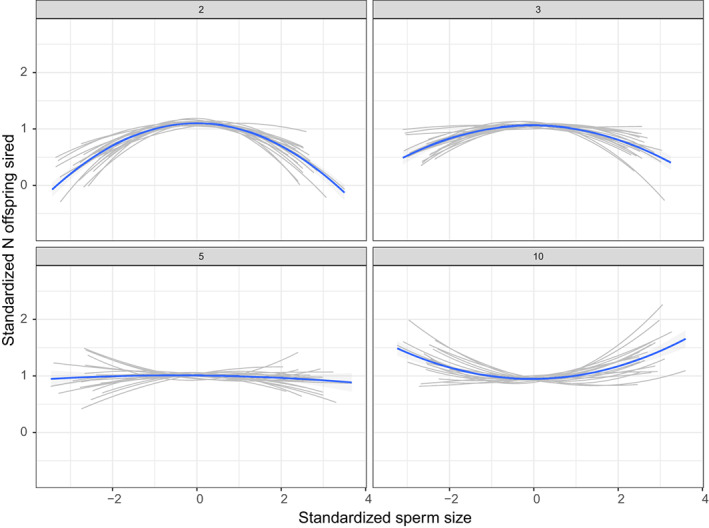
Examples of selection gradients showing the relationship between standardized sperm size and male reproductive success (standardized number of offspring sired), for 20 randomly selected populations with the SD for among‐female variation in preference = 2. Each panel shows a different level of number of copulation partners. The male trait had a standard deviation of 1 in all treatments. Grey lines show 20 randomly selected individual populations and the blue line shows the overall pattern within these 20 populations.

**FIGURE 3:**
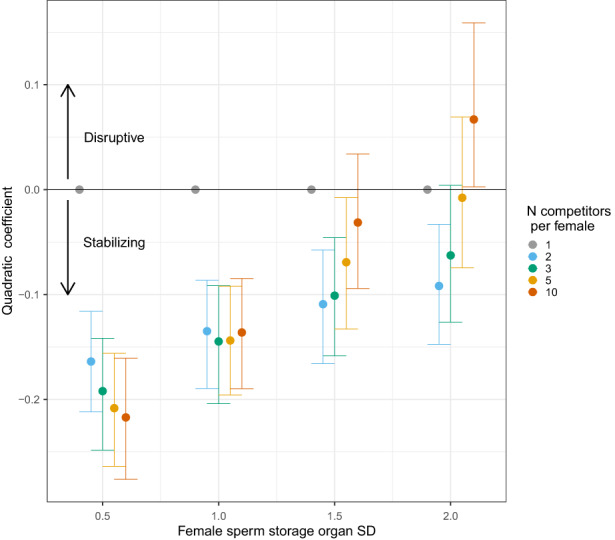
Values of the quadratic selection gradient for all simulation conditions. Colours indicate the number of copulation partners. A value of 0 (heavy horizontal line) indicates no quadratic selection; negative values indicate stabilizing selection; and positive values indicate disruptive selection. Results include 1000 replicates per simulation condition. Bars show the 95% quantiles calculated from the 1000 replicate simulations, and points are the medians.

The value of the quadratic selection gradient term depended on among‐female variation in sperm storage organs (*η* ^2^ = 0.38), number of copulation partners (*η* ^2^ = 0.31) and the interaction between the two variables (*η* ^2^ = 0.20; Figures 2 and 3). Quadratic selection estimates changed dramatically with variation in female sperm storage organs when the number of copulation partners was high (10 partners), going from strongly stabilizing to disruptive (the 95% quantiles excluded 0 with SD in female sperm storage organs equal to two; Figure 3). However, when females copulated only two or three times, selection was stabilizing, and it was only slightly less stabilizing when variation in female sperm storage organs was higher (Figure 3). There was no selection on sperm when the female copulated with only one male (Figure 3). Interestingly, increasing promiscuity had no impact on the strength of stabilizing selection where the SD for female sperm storage organs was 1, and in some conditions, there was overall no selection on sperm despite high promiscuity (with 5 copulation partners with SD in female sperm storage organs 2, Figures 2 and 3; and with 10 copulation partners and SD in female sperm storage organs 1.5, Figure 3). Overall patterns were similar in the additional simulation conditions tested (see Appendix S1).

### 3.2 Analytical model

From Equation (11), we see that the shape and intercept of *R*(*y*) is determined by the expression $-\sigma_{F}^{2}+\sigma_{M}^{2}+\sigma_{U}^{2}$. In particular, if $\sigma_{F}^{2}<\sigma_{M}^{2}+\sigma_{U}^{2}$, *R*(*y*) is bell‐shaped and has *R*(0) > 1 as its maximum value, indicating that males with average sperm size gain greater fertilization success than would be expected given their frequency in the population. This implies stabilizing selection. If $\sigma_{F}^{2}>\sigma_{M}^{2}+\sigma_{U}^{2}$, *R*(*y*) is U‐shaped and *R*(0) < 1, indicating that males with average sperm size gain less fertilization success than expected and implying disruptive selection. No selection is expected where $\sigma_{F}^{2}=\sigma_{M}^{2}+\sigma_{U}^{2}$ as this results in the constant function *R*(*y*) = 1. We evaluated whether this result agreed with model results by arbitrarily choosing several sets of values for the three variances that should give no quadratic selection (see Appendix, Table S1).

## 4 DISCUSSION

Stabilizing selection on sperm size when there is less variation in female sperm storage organs than in sperm is intuitive: all females' sperm storage organs are best fit by sperm with a phenotype close to the male population mean. Disruptive selection when female sperm storage organs are more variable than sperm is similarly intuitive: many females would be best fit by sperm outside the sperm size distribution, thus the most extreme males in the population obtain high fertilization success after copulating with a matching female (Millan et al., 2020; Van Doorn et al., 2001; van Doorn & Weissing, 2002). Our analytical model indicates that the change from stabilizing to disruptive selection should occur when the among‐female sperm storage organ variance is greater than the sum of the among‐male variance in sperm size and the variance parameter in the female preference function (Equation 1, which we do not vary in the main simulations). Our simulation results, however, show stabilizing selection under more conditions than expected, also compared to previous models where females sampled a large subset of males (Millan et al., 2020). Specifically, when the number of copulation partners is low, stabilizing selection can occur even with high among‐female variation in sperm storage organs. We suggest that this stabilizing selection occurs because males with relatively extreme sperm values are unlikely to copulate with females with a matching sperm storage organ, and their fertilization advantage when they do achieve these matching copulations is insufficient to offset the rarity of the copulations. The importance of sampling number is also evident in the empirical literature, where mating preferences are expressed more strongly in studies where individuals can choose among two mating options, compared to studies where individuals have a single option and can mate or not (Dougherty & Shuker, 2015).

### 4.1 Implications for sperm evolution

This study shows that variation in cryptic female preferences (here exemplified by variation in sperm storage organ size) and variation in number of copulation partners each can have a strong impact on the strength, or even shape, of selection on sperm morphology. Perhaps surprisingly, there are conditions (i.e. where sperm and sperm storage organ variation are equal) where number of copulation partners does not impact the strength of selection, and there are conditions where no selection on sperm is expected even when there are a large number of copulation partners. It is difficult to know which combination of variables is likely to be most biologically relevant, since copulation behaviour is difficult to observe in the wild and variation in female genital morphology remains understudied relative to male genital traits (Ah‐King et al., 2014; Orbach, 2022), despite recent effort (e.g. Brennan, 2022; Orbach et al., 2018; Simmons & Fitzpatrick, 2019). However, we can draw some generalizations. With low levels of promiscuity (2–3 copulation partners), selection is expected to be stabilizing, and it is similar across levels of variation in female sperm storage organ size. In contrast, with high numbers of copulation partners (≥10), selection on sperm is stabilizing, null, or disruptive, depending on the level of variation in the female sperm storage organs.

#### 4.1.1 Low to moderate promiscuity systems

For many species, we suspect that the number of copulation partners is low enough that stabilizing selection is broadly expected. Inferences of copulation rate from paternity patterns suggest in socially monogamous passerine birds that females on average copulate with fewer than 3 males (Brommer et al., 2007, 2010; Cramer, Kaiser, et al., 2020). Genotyping remnants of stored sperm in the female reproductive tract indicates that mean number of mates is between 2 and 6 for several invertebrates (including butterflies, crickets and beetles; Gage, 1994; Simmons et al., 2007; Simmons & Beveridge, 2010; Turnell & Shaw, 2015) and for sharks (Rowley, Daly‐Engel, et al., 2019). In such species, assuming heritability of sperm morphology (reviewed by Edme et al., 2019), the sperm‐female fit function modelled here would then often be expected to erode variation in sperm morphology over time. Why, then, are sperm cells still variable, and why does the level of variability correlate with promiscuity rates?

Stabilizing selection imposed by the need to fit the female's sperm storage organs may be countered by diverse other selective pressures. For example, different sperm morphology may confer a fertilization advantage depending on whether the sperm are the first‐inseminated (i.e. in a defensive position relative to competitors) or are later‐inseminated (in an offensive role) (Calhim et al., 2011; Clark et al., 1995), as well as on the length of the sperm of previous copulation partners (Lüpold et al., 2020). The most advantageous sperm morphology may also depend on the phenotype of the male himself (Ålund et al., 2018). Sperm morphology may correlate with other ejaculate traits that are also under selection, such as sperm number and sperm swimming speed, resulting in complex multivariate selection patterns (Fitzpatrick et al., 2012; Lüpold, Manier, et al., 2012; Snook, 2005). Sperm morphology may correlate with pre‐copulatory traits under selection (e.g. Simmons et al., 2017), creating indirect selection on sperm morphology (Cramer, 2021). Finally, selection for genetically compatible sperm (Bretman et al., 2009; Fossøy et al., 2008; Gasparini & Pilastro, 2011; Rekdal et al., 2019; Simmons et al., 2006) is expected to be independent of sperm morphology, since it depends on the genotypes of the male and female. As these examples show, it is most appropriate to consider the sperm storage organ fit as one component of a complex selective landscape.

At an ontogenetic level, variation in sperm morphology may arise due to various environmental factors, including but not limited to age (e.g. Cramer, Krauss, et al., 2020), seasonal changes in sperm morphology (Cramer et al., 2013; Edme et al., 2019; Lüpold, Birkhead, & Westneat, 2012), larval rearing conditions and timing (Vermeulen et al., 2009), differences in the social environment as an adult (Immler et al., 2010; Rojas Mora et al., 2018) and condition‐dependence of sperm phenotypes (which has been documented in some studies but is not generally expected; Macartney et al., 2019).

Persistence of variation in sperm morphology may also depend on the genetic and genomic underpinnings of the trait. In zebra finches, for example, a genomic inversion on the sex chromosome allows many loci to act as a super gene influencing sperm morphology (Kim et al., 2017), and this supergene shows heterozygote advantage that could sustain genetic variation over time (Knief et al., 2017). Maternal genetic effects on sperm traits have been found in several studies (e.g. Froman et al., 2002; Morrow & Gage, 2001; Ward, 2000). If the genes causing these maternal effects are X‐linked or on the mitochondria, they may be protected to some extent from selection acting on the sperm phenotype (Gemmell et al., 2004). Genetic underpinnings of sperm morphology are poorly known for most species, although substantial heritability of sperm morphology indicates strong genetic effects (reviewed in Edme et al., 2019). However, heritability is less directly relevant to how quickly a trait is expected to evolve in response to selection than is evolvability (Hansen et al., 2011). Evolvability for sperm morphological traits is comparable to values for other linear trait measurements (median 0.1% for linear traits in Hansen et al., 2011; range for total sperm length 0.02%–0.26% in Edme et al., 2019, recalculated from CV_A_ to I_A_ for comparability to Hansen et al., 2011). Notably, sperm length and sperm storage organ length may be positively (Lupold et al., 2016) or negatively (Simmons & Kotiaho, 2007) genetically correlated, which would have important implications for how these traits co‐evolve.

The above examples may help to explain why sperm remain variable despite stabilizing selection, but they do not immediately explain the among‐species correlation between promiscuity level and intraspecific sperm morphological variation. Here, at least for social monogamy with extra‐pair paternity and other taxa where promiscuity level primarily reflects differences in the risk, rather than the intensity, of sperm competition (sensu Parker et al., 1996), the among‐species pattern may partly reflect the proportion of non‐promiscuous females in the population. Some females may copulate only with their social mate, for example due to an optimal social mate choice or successful mate‐guarding by that male (Westneat et al., 1990; Westneat & Stewart, 2003) and, therefore, exert no selection on sperm morphology (assuming that all her eggs get fertilized; Vuarin et al., 2019). Note that frequent copulations by social males only have a marginal impact on selection on sperm morphology (Figures S2 and S3). If we assume that monogamous females exert no selection on sperm and females copulating with 2–3 males exert stabilizing selection (as indicated in the model), then the total strength of stabilizing selection should depend on the proportion of monogamous versus promiscuous females. Assuming that the proportion of monogamous females is lower in species with higher extra‐pair paternity rates, we then can expect stronger overall stabilizing selection on sperm in those species. However, it is unclear to what extent variation in extra‐pair paternity rates actually reflects variation in the proportion of monogamous females, as field studies indicate that a substantial number of extra‐pair copulations fail to result in any fertilizations (Fossøy et al., 2006; Michl et al., 2002). Regardless, strong stabilizing selection due to high proportions of females obtaining 2–3 copulation partners may result in faster evolution of sperm in these lineages (as seen in Rowe et al., 2015), if mean sperm storage organ size becomes different from mean sperm size, for example due to genetic drift (Figures S8–S11).

#### 4.1.2 High‐promiscuity systems

For some groups, for example some eusocial insects, the number of copulation partners can be quite high (Tarpy et al., 2004) (although the sperm‐sperm storage organ fit mechanism modelled here appears unlikely to apply in, e.g. honey bees, Snodgrass, 1910). In systems with high numbers of copulation partners (i.e. 10 or more), we expect the shape of selection to depend strongly on the degree of variation in female sperm storage organs, ranging from stabilizing to disruptive selection, although the myriad other factors influencing sperm variation discussed above may also be at play in high‐promiscuity systems. The combination of high promiscuity and high variation in female sperm storage organs creates an expectation of disruptive sexual selection, which, in turn, can play a role in the splitting of lineages to form separate species (Lande, 1981; van Doorn & Weissing, 2002; Weissing et al., 2011; see also Van Doorn et al., 2001; Howard et al., 2009).

### 4.2 Implications for previous work on mate choice

Our observation that limited mate sampling causes stabilizing selection even with substantial among‐female variation in the preferred male phenotype has important implications for interpreting previous models of sympatric speciation. Previous models have highlighted a broad among‐female distribution in the preferred male phenotype as a key element in generating disruptive selection on male traits, as one step that can lead to sympatric speciation (Higashi et al., 1999; Van Doorn et al., 2001; van Doorn & Weissing, 2002; Weissing et al., 2011). Our results suggest that disruptive selection will occur under more limited circumstances than was previously appreciated, since females generally are expected to be somewhat limited in the number of males they can sample (Jennions & Petrie, 1997). We thus support Servedio and Boughman's (2017) assertion that novel insights may be obtained in the sympatric speciation literature by further exploring closed‐ended preference functions and limited female searches, similar to what we have simulated here.

As expected from previous models (Gomulkiewicz, 1991; Janetos, 1980; Muniz & Machado, 2018), increasing the number of partners could increase the strength of selection. However, when the most‐preferred male trait value varied among females, changes in sampling can change the shape of selection and not only its strength. Moreover, in some cases, higher sampling can lead to weaker selection. We further find that selection is generally weaker when paternity is shared within each batch of offspring, compared to when the best fit male sires all offspring (Figure S13). Models of mate choice should, therefore, use realistic values for number of males sampled and number of males succeeding (in copulating or fertilizing) to obtain the most biologically relevant results.

## 5 CONCLUSIONS

Despite broad interest in sperm morphology, relatively few studies have evaluated selection on sperm morphology in the wild (Lüpold & Pitnick, 2018), and even fewer have evaluated the presence and effect of variation in female sperm storage organs. Under our model where the sperm storage organs bias paternity success towards sperm of a similar size, the level of variation in the female sperm storage organs determines whether selection on sperm is stabilizing or disruptive for highly promiscuous species, whereas selection is stabilizing for species with only 2–3 copulations per female reproductive bout. These results may also be more broadly relevant where morphological fit between male and female is important, for example, genital coevolution for species where copulation involves intromission (Brennan, 2016).

## AUTHOR CONTRIBUTIONS

**Emily R. A. Cramer:** Conceptualization (equal); formal analysis (lead); methodology (equal); software (lead); writing – original draft (lead); writing – review and editing (equal). **Zelealem B. Yilma:** Formal analysis (lead); methodology (equal); software (supporting); writing – review and editing (equal). **Jan T. Lifjeld:** Conceptualization (equal); funding acquisition (lead); methodology (equal); writing – review and editing (equal).

## CONFLICT OF INTEREST

The authors declare they have no conflicts of interest.

## Supporting information


Appendix S1

## Data Availability

Code required to replicate main simulation results has been archived on Zenodo (https://doi.org/10.5281/zenodo.7128886).

## References

[jeb14120-bib-0001] Ah‐King, M. , Barron, A. B. , & Herberstein, M. E. (2014). Genital evolution: Why are females still understudied? PLoS Biology, 12, 1–7. 10.1371/journal.pbio.1001851 PMC4011675 24802812

[jeb14120-bib-0002] Ålund, M. , Schmiterlöw, S. P. , McFarlane, S. E. , & Qvarnström, A. (2018). Optimal sperm length for high siring success depends on forehead patch size in collared flycatchers. Behavioral Ecology, 29, 1436–1443.

[jeb14120-bib-0003] Amitin, E. G. , & Pitnick, S. (2007). Influence of developmental environment on male‐ and female‐mediated sperm precedence in *Drosophila melanogaster* . Journal of Evolutionary Biology, 20, 381–391. 17210031 10.1111/j.1420-9101.2006.01184.x

[jeb14120-bib-0004] Berger, D. , Bauerfeind, S. S. , Blanckenhorn, W. U. , & Schäfer, M. A. (2011). High temperatures reveal cryptic genetic variation in a polymorphic female sperm storage organ. Evolution, 65, 2830–2842. 21967425 10.1111/j.1558-5646.2011.01392.x

[jeb14120-bib-0005] Birkhead, T. R. , Chaline, N. , Biggins, J. D. , Burke, T. , & Pizzari, T. (2004). Nontransitivity of paternity in a bird. Evolution, 58, 416–420. 15068357

[jeb14120-bib-0006] Bjork, A. , Starmer, W. T. , Higginson, D. M. , Rhodes, C. J. , & Pitnick, S. (2007). Complex interactions with females and rival males limit the evolution of sperm offence and defence. Proceedings of the Royal Society B: Biological Sciences, 274, 1779–1788. 10.1098/rspb.2007.0293 PMC2493577 17507332

[jeb14120-bib-0007] Brennan, P. L. R. (2016). Studying genital coevolution to understand intromittent organ morphology. Integrative and Comparative Biology, 56, 669–681. 27252198 10.1093/icb/icw018

[jeb14120-bib-0008] Brennan, P. L. R. (2022). Evolution and morphology of genitalia in female amniotes. Integrative and Comparative Biology, 62, 521–532. 10.1093/icb/icac115 35798011

[jeb14120-bib-0009] Bretman, A. , Newcombe, D. , & Tregenza, T. (2009). Promiscuous females avoid inbreeding by controlling sperm storage. Molecular Ecology, 18, 3340–3345. 19694961 10.1111/j.1365-294X.2009.04301.x

[jeb14120-bib-0010] Briskie, J. V. (1996). Spatiotemporal of sperm storage and last‐male patterns in birds sperm precedence. Functional Ecology, 10, 375–383.

[jeb14120-bib-0011] Briskie, J. V. , & Montgomerie, R. (1992). Sperm size and sperm competition in birds. Proceedings of the Royal Society B: Biological Sciences, 247, 89–95. 10.1098/rspb.1992.0013 1349186

[jeb14120-bib-0012] Brommer, J. E. , Alho, J. S. , Biard, C. , Chapman, J. R. , Charmantier, A. , Dreiss, A. , Hartley, I. R. , Hjernquist, M. B. , Kempenaers, B. , Komdeur, J. , Laaksonen, T. , Lehtonen, P. K. , Lubjuhn, T. , Patrick, S. C. , Rosivall, B. , Tinbergen, J. M. , van der Velde, M. , van Oers, K. , Wilk, T. , & Winkel, W. (2010). Passerine extrapair mating dynamics: A Bayesian modeling approach comparing four species. The American Naturalist, 176, 178–187. 10.1086/653660 20528475

[jeb14120-bib-0013] Brommer, J. E. , Korsten, P. , Bouwman, K. M. , Berg, M. L. , & Komdeur, J. (2007). Is extrapair mating random? On the probability distribution of extrapair young in avian broods. Behavioral Ecology, 18, 895–904.

[jeb14120-bib-0014] Brouwer, L. , & Griffith, S. C. (2019). Extra‐pair paternity in birds. Molecular Ecology, 28, 4864–4882. 31587397 10.1111/mec.15259 PMC6899757

[jeb14120-bib-0015] Calhim, S. , Double, M. C. , Margraf, N. , Birkhead, T. R. , & Cockburn, A. (2011). Maintenance of sperm variation in a highly promiscuous wild bird. PLoS One, 6, e28809. 22194918 10.1371/journal.pone.0028809 PMC3240631

[jeb14120-bib-0016] Calhim, S. , Immler, S. , & Birkhead, T. R. (2007). Postcopulatory sexual selection is associated with reduced variation in sperm morphology. PLoS One, 2, e413. 17476335 10.1371/journal.pone.0000413 PMC1855076

[jeb14120-bib-0017] Clark, A. G. (2002). Sperm competition and the maintenance of polymorphism. Heredity, 88, 148–153. 11932773 10.1038/sj.hdy.6800019

[jeb14120-bib-0018] Clark, A. G. , Aguade, M. , Prout, T. , Harshman, L. G. , & Langley, C. H. (1995). Variation in sperm displacement and its association with accessory gland protein loci in *Drosophila melanogaster* . Genetics, 139, 189–201. 7705622 10.1093/genetics/139.1.189 PMC1206317

[jeb14120-bib-0019] Cramer, E. R. A. (2021). Measuring pre‐ and post‐copulatory sexual selection and their interaction in socially monogamous species with extra‐pair paternity. Cell, 10, 620. 10.3390/cells10030620 PMC7999480 33799610

[jeb14120-bib-0020] Cramer, E. R. A. , Kaiser, S. A. , Webster, M. S. , & Ryder, T. B. (2020). Common field data limitations can substantially bias sexual selection metrics. The American Naturalist, 196, 180–196. 10.1086/709547 32673091

[jeb14120-bib-0021] Cramer, E. R. A. , Krauss, N. , Rowlison, T. , & Comizzoli, P. (2020). Sperm morphology and male age in black‐throated blue warblers, an ecological model system. Animals, 10, 1175. 32664407 10.3390/ani10071175 PMC7401543

[jeb14120-bib-0022] Cramer, E. R. A. , Laskemoen, T. , Eroukhmanoff, F. , Haas, F. , Hermansen, J. S. , Lifjeld, J. T. , Rowe, M. , Sætre, G. P. , & Johnsen, A. (2014). Testing a post‐copulatory pre‐zygotic reproductive barrier in a passerine species pair. Behavioral Ecology and Sociobiology, 68, 1133–1144.

[jeb14120-bib-0023] Cramer, E. R. A. , Laskemoen, T. , Kleven, O. , & Lifjeld, J. T. (2013). Sperm length variation in house wrens *Troglodytes aedon* . Journal für Ornithologie, 154, 129–138.

[jeb14120-bib-0024] Cramer, E. R. A. , Stensrud, E. , Marthinsen, G. , Hogner, S. , Johannessen, L. E. , Laskemoen, T. , Eybert, M. C. , Slagsvold, T. , Lifjeld, J. T. , & Johnsen, A. (2016). Sperm performance in conspecific and heterospecific female fluid. Ecology and Evolution, 6, 1363–1377. 26855769 10.1002/ece3.1977 PMC4733106

[jeb14120-bib-0025] den Boer, S. P. A. , Baer, B. , & Boomsma, J. J. (2010). Seminal fluid mediates ejaculate competition in social insects. Science, 327, 1506–1509. 20299595 10.1126/science.1184709

[jeb14120-bib-0026] Dieckmann, U. , & Doebeli, M. (1999). On the origin of species by sympatric speciation. Nature, 400, 354–357. 10432112 10.1038/22521

[jeb14120-bib-0027] Dougherty, L. R. , & Shuker, D. M. (2015). The effect of experimental design on the measurement of mate choice: A meta‐analysis. Behavioral Ecology, 26, 311–319.

[jeb14120-bib-0028] Dybas, L. K. , & Dybas, H. S. (1981). Coadaptation and taxonomic differentiation of sperm and spermathecae in featherwing beetles. Evolution, 35, 168–174. 28563443 10.1111/j.1558-5646.1981.tb04869.x

[jeb14120-bib-0029] Eady, P. E. , & Brown, D. V. (2017). Male–female interactions drive the (Un)repeatability of copula duration in an insect. Royal Society Open Science, 4, 1–7. 28386449 10.1098/rsos.160962 PMC5367291

[jeb14120-bib-0030] Eberhard, W. (1996). Female control: Sexual selection by cryptic female choice. Princeton University Press.

[jeb14120-bib-0031] Edme, A. , Zobač, P. , Korsten, P. , Albrecht, T. , Schmoll, T. , & Krist, M. (2019). Moderate heritability and low evolvability of sperm morphology in a species with high risk of sperm competition, the collared flycatcher *Ficedula albicollis* . Journal of Evolutionary Biology, 32, 205–217. 30449037 10.1111/jeb.13404

[jeb14120-bib-0032] Farrow, R. A. , Deeming, D. C. , & Eady, P. E. (2022). Male and female developmental temperature modulate post‐copulatory interactions in a beetle. Journal of Thermal Biology, 103, 103155. 35027191 10.1016/j.jtherbio.2021.103155

[jeb14120-bib-0033] Fitzpatrick, J. L. , & Baer, B. (2011). Polyandry reduces sperm length variation in social insects. Evolution, 65, 3006–3012. 21967440 10.1111/j.1558-5646.2011.01343.x

[jeb14120-bib-0034] Fitzpatrick, J. L. , Simmons, L. W. , & Evans, J. P. (2012). Complex patterns of multivariate selection on the ejaculate of a broadcast spawning marine invertebrate. Evolution, 66, 2451–2460. 22834744 10.1111/j.1558-5646.2012.01627.x

[jeb14120-bib-0035] Fossøy, F. , Johnsen, A. , & Lifjeld, J. T. (2006). Evidence of obligate female promiscuity in a socially monogamous passerine. Behavioral Ecology and Sociobiology, 60, 255–259.

[jeb14120-bib-0036] Fossøy, F. , Johnsen, A. , & Lifjeld, J. T. (2008). Multiple genetic benefits of female promiscuity in a socially monogamous passerine. Evolution, 62, 145–156. 18053073 10.1111/j.1558-5646.2007.00284.x

[jeb14120-bib-0037] Froman, D. P. , Pizzari, T. , Feltmann, A. J. , Castillo‐Juarez, H. , & Birkhead, T. R. (2002). Sperm mobility: Mechanisms of fertilizing efficiency, genetic variation and phenotypic relationship with male status in the domestic fowl, *Gallus gallus domesticus* . Proceedings of the Royal Society B: Biological Sciences, 269, 607–612. 10.1098/rspb.2001.1925 PMC1690926 11916477

[jeb14120-bib-0038] Gage, M. J. G. (1994). Associations between body size, mating pattern, testis size and sperm lengths across butterflies. Proceedings of the Royal Society B: Biological Sciences, 258, 247–254.

[jeb14120-bib-0039] García‐González, F. , & Simmons, L. W. (2007). Shorter sperm confer higher competitive fertilization success. Evolution, 61, 816–824. 17439614 10.1111/j.1558-5646.2007.00084.x

[jeb14120-bib-0040] Gasparini, C. , & Pilastro, A. (2011). Cryptic female preference for genetically unrelated males is mediated by ovarian fluid in the guppy. Proceedings of the Royal Society B: Biological Sciences, 278, 2495–2501. 10.1098/rspb.2010.2369 PMC3125622 21227973

[jeb14120-bib-0041] Gemmell, N. J. , Metcalf, V. J. , & Allendorf, F. W. (2004). Mother's curse: The effect of mtDNA on individual fitness and population viability. Trends in Ecology & Evolution, 19, 238–244. 16701262 10.1016/j.tree.2004.02.002

[jeb14120-bib-0042] Gomulkiewicz, R. (1991). Limited female choice and the evolution of secondary sexual characters. The American Naturalist, 138, 1396–1407.

[jeb14120-bib-0043] Hansen, T. F. , Pélabon, C. , & Houle, D. (2011). Heritability is not evolvability. Evolutionary Biology, 38, 258–277.

[jeb14120-bib-0044] Hellriegel, B. , & Bernasconi, G. (2000). Female‐mediated differential sperm storage in a fly with complex spermathecae, *Scatophaga stercoraria* . Animal Behaviour, 59, 311–317. 10675253 10.1006/anbe.1999.1308

[jeb14120-bib-0045] Hemmings, N. , Bennison, C. , & Birkhead, T. R. (2016). Intra‐ejaculate sperm selection in female zebra finches. Biology Letters, 12, 4–7. 10.1098/rsbl.2016.0220 PMC4938051 27277953

[jeb14120-bib-0046] Hemmings, N. , Bennison, C. , & Birkhead, T. R. (2018). Data from: Intra‐ejaculate sperm selection in female zebra finches. *Dryad Dataset*. 10.5061/dryad.vc7ss PMC4938051 27277953

[jeb14120-bib-0047] Hemmings, N. , & Birkhead, T. (2017). Differential sperm storage by female zebra finches *Taeniopygia guttata* . Proceedings of the Royal Society B: Biological Sciences, 284, 20171032. 10.1098/rspb.2017.1032 PMC5563812 28814657

[jeb14120-bib-0048] Higashi, M. , Takimoto, G. , & Yamamura, N. (1999). Sympatric speciation by sexual selection. Nature, 402, 523–526. 10591210 10.1038/990087

[jeb14120-bib-0049] Higginson, D. M. , Miller, K. B. , Segraves, K. A. , & Pitnick, S. (2012). Female reproductive tract form drives the evolution of complex sperm morphology. Proceedings of the National Academy of Sciences of the United States of America, 109, 4538–4543. 22323584 10.1073/pnas.1111474109 PMC3311347

[jeb14120-bib-0050] Hosken, D. J. , Garner, T. W. J. , & Ward, P. I. (2001). Sexual conflict selects for male and female reproductive characters. Current Biology, 11, 489–493. 11412998 10.1016/s0960-9822(01)00146-4

[jeb14120-bib-0051] Howard, D. J. , Palumbi, S. R. , Birge, L. M. , & Manier, M. K. (2009). Sperm and speciation. In T. R. Birkhead , D. J. Hosken , & S. Pitnick (Eds.), Sperm biology: An evolutionary perspective (pp. 367–404). Academic Press.

[jeb14120-bib-0052] Immler, S. , Pryke, S. R. , Birkhead, T. R. , & Griffith, S. C. (2010). Pronounced within‐individual plasticity in sperm morphometry across social environments. Evolution, 64, 1634–1643. 20015235 10.1111/j.1558-5646.2009.00924.x

[jeb14120-bib-0053] Janetos, A. C. (1980). Strategies of female mate choice: A theoretical analysis. Behavioral Ecology and Sociobiology, 7, 107–112.

[jeb14120-bib-0054] Jennions, M. D. , & Petrie, M. (1997). Variation in mate choice and mating preferences: A review of causes and consequences. Biological Reviews, 72, 283–327. 9155244 10.1017/s0006323196005014

[jeb14120-bib-0055] Jennions, M. D. , & Petrie, M. (2000). Why do females mate multiply? A review of the genetic benefits. Biological Reviews, 75, 21–64. 10740892 10.1017/s0006323199005423

[jeb14120-bib-0056] Kahrl, A. F. , Kustra, M. C. , Reedy, A. M. , Bhave, R. S. , Seears, H. A. , Warner, D. A. , & Cox, R. M. (2021). Selection on sperm count, but not on sperm morphology or velocity, in a wild population of anolis lizards. Cell, 10, 1–13. 10.3390/cells10092369 PMC8464841 34572018

[jeb14120-bib-0057] Kahrl, A. F. , Snook, R. R. , & Fitzpatrick, J. L. (2021). Fertilization mode drives sperm length evolution across the animal tree of life. Nature Ecology and Evolution, 5, 1153–1164. 34155385 10.1038/s41559-021-01488-y

[jeb14120-bib-0058] Kim, K. W. , Bennison, C. , Hemmings, N. , Brookes, L. , Hurley, L. L. , Griffith, S. C. , Burke, T. , Birkhead, T. R. , & Slate, J. (2017). A sex‐linked supergene controls sperm morphology and swimming speed in a songbird. Nature Ecology and Evolution, 1, 1168–1176. 29046578 10.1038/s41559-017-0235-2

[jeb14120-bib-0059] Knief, U. , Forstmeier, W. , Pei, Y. , Ihle, M. , Wang, D. , Martin, K. , Opatová, P. , Albrechtová, J. , Wittig, M. , Franke, A. , Albrecht, T. , & Kempenaers, B. (2017). A sex‐chromosome inversion causes strong overdominance for sperm traits that affect siring success. Nature Ecology and Evolution, 1, 1177–1184. 29046576 10.1038/s41559-017-0236-1

[jeb14120-bib-0060] Lande, R. (1981). Models of speciation by sexual selection on polygenic traits. Proceedings of the National Academy of Sciences of the United States of America, 78, 3721–3725. 16593036 10.1073/pnas.78.6.3721 PMC319643

[jeb14120-bib-0061] Lande, R. , & Arnold, S. J. (1983). The measurement of selection on correlated characters. Evolution, 37, 1210–1226. 28556011 10.1111/j.1558-5646.1983.tb00236.x

[jeb14120-bib-0062] Lifjeld, J. T. , Laskemoen, T. , Kleven, O. , Albrecht, T. , & Robertson, R. J. (2010). Sperm length variation as a predictor of extrapair paternity in passerine birds. PLoS One, 5, e13456. 20976147 10.1371/journal.pone.0013456 PMC2956655

[jeb14120-bib-0063] Løvlie, H. , Gillingham, M. A. F. , Worley, K. , Pizzari, T. , & Richardson, D. S. (2013). Cryptic female choice favours sperm from major histocompatibility complex‐dissimilar males. Proceedings of the Royal Society B: Biological Sciences, 280, 20131296. 10.1098/rspb.2013.1296 PMC3768299 24004935

[jeb14120-bib-0064] Lüdecke, D. (2021). *sjstats: Statistical functions for regression models* (Version 0.18.1).

[jeb14120-bib-0065] Lüpold, S. , Birkhead, T. R. , & Westneat, D. F. (2012). Seasonal variation in ejaculate traits of male red‐winged blackbirds (*Agelaius phoeniceus*). Behavioral Ecology and Sociobiology, 66, 1607–1617.

[jeb14120-bib-0066] Lüpold, S. , Manier, M. K. , Berben, K. S. , Smith, K. J. , Daley, B. D. , Buckley, S. H. , Belote, J. M. , & Pitnick, S. (2012). How multivariate ejaculate traits determine competitive fertilization success in *Drosophila melanogaster* . Current Biology, 22, 1667–1672. 22840512 10.1016/j.cub.2012.06.059

[jeb14120-bib-0067] Lupold, S. , Manier, M. K. , Puniamoorthy, N. , Schoff, C. , Starmer, W. T. , Luepold, S. H. B. , Belote, J. M. , & Pitnick, S. (2016). How sexual selection can drive the evolution of costly sperm ornamentation. Nature, 533, 535–538. 27225128 10.1038/nature18005

[jeb14120-bib-0068] Lüpold, S. , & Pitnick, S. (2018). Sperm form and function: What do we know about the role of sexual selection? Reproduction, 155, R229–R243. 29459400 10.1530/REP-17-0536

[jeb14120-bib-0069] Lüpold, S. , Pitnick, S. , Berben, K. S. , Blengini, C. S. , Belote, J. M. , & Manier, M. K. (2013). Female mediation of competitive fertilization success in Drosophila melanogaster. Proceedings of the National Academy of Sciences of the United States of America, 110, 10693–10698. 23757499 10.1073/pnas.1300954110 PMC3696778

[jeb14120-bib-0070] Lüpold, S. , Reil, J. B. , Manier, M. K. , Zeender, V. , Belote, J. M. , & Pitnick, S. (2020). How female × male and male × male interactions influence competitive fertilization in *Drosophila melanogaster* . Evolution Letters, 4, 416–429. 33014418 10.1002/evl3.193 PMC7523561

[jeb14120-bib-0071] Macartney, E. L. , Crean, A. J. , Nakagawa, S. , & Bonduriansky, R. (2019). Effects of nutrient limitation on sperm and seminal fluid: A systematic review and meta‐analysis. Biological Reviews, 94, 1722–1739. 31215758 10.1111/brv.12524

[jeb14120-bib-0072] Manier, M. K. , Belote, J. M. , Berben, K. S. , Novikov, D. , Stuart, W. T. , & Pitnick, S. (2010). Resolving mechanisms of competitive fertiliation success in *Drosophila melanogaster* . Science, 328, 354–357. 20299550 10.1126/science.1187096

[jeb14120-bib-0073] Mendonca, T. , Cadby, A. J. , & Hemmings, N. (2019). Sperm gatekeeping: 3D Imaging reveals a constricted entrance to zebra finch sperm storage tubules. Biophysical Journal, 117, 2180–2187. 31733725 10.1016/j.bpj.2019.10.038 PMC6895713

[jeb14120-bib-0074] Michl, G. , Török, J. , Griffith, S. C. , & Sheldon, B. C. (2002). Experimental analysis of sperm competition mechanisms in a wild bird population. Proceedings of the National Academy of Sciences of the United States of America, 99, 5466–5470. 11943862 10.1073/pnas.082036699 PMC122792

[jeb14120-bib-0075] Millan, C. H. , Machado, G. , & Muniz, D. G. (2020). Within‐population variation in female mating preference affects the opportunity for sexual selection and the evolution of male traits, but things are not as simple as expected. Journal of Evolutionary Biology, 33, 1579–1592. 32871625 10.1111/jeb.13694

[jeb14120-bib-0076] Miller, G. T. , & Pitnick, S. (2002). Sperm‐female coevolution in *Drosophila* . Science, 298, 1230–1233. 12424377 10.1126/science.1076968

[jeb14120-bib-0077] Miller, G. T. , Starmer, W. T. , & Pitnick, S. (2003). Quantitative genetic analysis of among‐population variation in sperm and female sperm‐storage organ length in *Drosophila mojavensis* . Genetical Research, 81, 213–220. 12929912 10.1017/s0016672303006190

[jeb14120-bib-0078] Morrow, E. H. , & Gage, M. J. G. (2001). Artificial selection and heritability of sperm length in *Gryllus bimaculatus* . Heredity, 87, 356–362. 11737282 10.1046/j.1365-2540.2001.00921.x

[jeb14120-bib-0079] Muniz, D. G. , & Machado, G. (2018). Mate sampling influences the intensity of sexual selection and the evolution of costly sexual ornaments. Journal of Theoretical Biology, 447, 74–83. 29567325 10.1016/j.jtbi.2018.03.026

[jeb14120-bib-0080] Orbach, D. N. (2022). Gender bias in the study of genital evolution: Females continue to receive less attention than males. Integrative and Comparative Biology, 62, 533–541. 10.1093/icb/icac012 35353194

[jeb14120-bib-0081] Orbach, D. N. , Hedrick, B. , Würsig, B. , Mesnick, S. L. , & Brennan, P. L. R. (2018). The evolution of genital shape variation in female cetaceans. Evolution, 72, 261–273. 29134627 10.1111/evo.13395

[jeb14120-bib-0082] Parker, G. (1970). Sperm competition and its evolutionary consequences in the insects. Biological Reviews, 45, 525–567.

[jeb14120-bib-0083] Parker, G. A. (1990). Sperm competition games: Raffles and roles. Proceedings of the Royal Society B: Biological Sciences, 242, 120–126.

[jeb14120-bib-0084] Parker, G. A. , Ball, M. A. , Stockley, P. , & Gage, M. J. G. (1996). Sperm competition games: Individual assessment of sperm competition intensity by group spawners. Proceedings of the Royal Society B: Biological Sciences, 263, 1291–1297.

[jeb14120-bib-0085] Pitnick, S. , Hosken, D. J. , & Birkhead, T. R. (2009). Sperm morphological diversity. In Sperm biology: An evolutionary perspective (pp. 69–150). Academic Press.

[jeb14120-bib-0086] R Development Core Team . (2020). R: A language and environment for statistical computing. R Foundation for statistical computing.

[jeb14120-bib-0087] Reinhart, M. , Carney, T. , Clark, A. G. , Fiumera, A. C. , & Markow, T. (2015). Characterizing male–female interactions using natural genetic variation in *Drosophila melanogaster* . Journal of Heredity, 106, 67–79. 25425680 10.1093/jhered/esu076 PMC4261835

[jeb14120-bib-0088] Rekdal, S. L. , Anmarkrud, J. A. , Lifjeld, J. T. , & Johnsen, A. (2019). Extra‐pair mating in a passerine bird with highly duplicated major histocompatibility complex class II: Preference for the golden mean. Molecular Ecology, 28, 5133–5144. 31614034 10.1111/mec.15273

[jeb14120-bib-0089] Richardson, J. T. E. (2011). Eta squared and partial eta squared as measures of effect size in educational research. Educational Research Review, 6, 135–147.

[jeb14120-bib-0090] Rojas Mora, A. , Meniri, M. , Ciprietti, S. , & Helfenstein, F. (2018). Is sperm morphology functionally related to sperm swimming ability? A case study in a wild passerine bird with male hierarchies. BMC Evolutionary Biology, 18, 1–11. 30231935 10.1186/s12862-018-1260-8 PMC6146611

[jeb14120-bib-0091] Rowe, M. , Albrecht, T. , Cramer, E. R. A. , Johnsen, A. , Laskemoen, T. , Weir, J. T. , & Lifjeld, J. T. (2015). Postcopulatory sexual selection is associated with accelerated evolution of sperm morphology. Evolution, 69, 1044–1052. 25655075 10.1111/evo.12620

[jeb14120-bib-0092] Rowley, A. , Locatello, L. , Kahrl, A. , Rego, M. , Boussard, A. , Garza‐Gisholt, E. , Kempster, R. M. , Collin, S. P. , Giacomello, E. , Follesa, M. C. , Porcu, C. , Evans, J. P. , Hazin, F. , Garcia‐Gonzalez, F. , Daly‐Engel, T. , Mazzoldi, C. , & Fitzpatrick, J. L. (2019). Sexual selection and the evolution of sperm morphology in sharks. Journal of Evolutionary Biology, 32, 1027–1035. 31250483 10.1111/jeb.13501

[jeb14120-bib-0093] Rowley, A. G. , Daly‐Engel, T. S. , & Fitzpatrick, J. L. (2019). Testes size increases with sperm competition risk and intensity in bony fish and sharks. Behavioral Ecology, 30, 364–371.

[jeb14120-bib-0094] Servedio, M. R. , & Boughman, J. W. (2017). The role of sexual selection in local adaptation and speciation. Annual Review of Ecology, Evolution, and Systematics, 48, 85–109.

[jeb14120-bib-0095] Simmons, L. W. , & Beveridge, M. (2010). The strength of postcopulatory sexual selection within natural populations of field crickets. Behavioral Ecology, 21, 1179–1185.

[jeb14120-bib-0096] Simmons, L. W. , Beveridge, M. , & Kennington, W. J. (2007). Polyandry in the wild: Temporal changes in female mating frequency and sperm competition intensity in natural populations of the tettigoniid *Requena verticalis* . Molecular Ecology, 16, 4613–4623. 17887967 10.1111/j.1365-294X.2007.03528.x

[jeb14120-bib-0097] Simmons, L. W. , Beveridge, M. , Wedell, N. , & Tregenza, T. (2006). Postcopulatory inbreeding avoidance by female crickets only revealed by molecular markers. Molecular Ecology, 15, 3817–3824. 17032276 10.1111/j.1365-294X.2006.03035.x

[jeb14120-bib-0098] Simmons, L. W. , & Fitzpatrick, J. L. (2019). Female genitalia can evolve more rapidly and divergently than male genitalia. Nature Communications, 10, 1–8. 10.1038/s41467-019-09353-0 PMC6428859 30899023

[jeb14120-bib-0099] Simmons, L. W. , & Kotiaho, J. S. (2007). Quantitative genetic correlation between trait and preference supports a sexually selected sperm process. Proceedings of the National Academy of Sciences of the United States of America, 104, 16604–16608. 17921254 10.1073/pnas.0704871104 PMC2034270

[jeb14120-bib-0100] Simmons, L. W. , Lovegrove, M. , & Almbro, M. (2014). Female effects, but no intrinsic male effects on paternity outcome in crickets. Journal of Evolutionary Biology, 27, 1644–1649. 24836498 10.1111/jeb.12418

[jeb14120-bib-0101] Simmons, L. W. , Lüpold, S. , & Fitzpatrick, J. L. (2017). Evolutionary trade‐off between secondary sexual traits and ejaculates. Trends in Ecology & Evolution, 32, 964–976. 29050795 10.1016/j.tree.2017.09.011

[jeb14120-bib-0102] Snodgrass, R. E. (1910). The anatomy of the honey bee. Government Printing Office.

[jeb14120-bib-0103] Snook, R. R. (2005). Sperm in competition: Not playing by the numbers. Trends in Ecology & Evolution, 20, 46–53. 16701340 10.1016/j.tree.2004.10.011

[jeb14120-bib-0104] Tarpy, D. R. , Nielsen, R. , & Nielsen, D. I. (2004). A scientific note on the revised estimates of effective paternity frequency in Apis. Insectes Sociaux, 51, 203–204.

[jeb14120-bib-0105] Turnell, B. R. , & Shaw, K. L. (2015). Polyandry and postcopulatory sexual selection in a wild population. Molecular Ecology, 24, 6278–6288. 26577698 10.1111/mec.13470

[jeb14120-bib-0106] Urbach, D. , Folstad, I. , & Rudolfsen, G. (2005). Effects of ovarian fluid on sperm velocity in Arctic charr (*Salvelinus alpinus*). Behavioral Ecology and Sociobiology, 57, 438–444.

[jeb14120-bib-0107] Van Doorn, G. S. , Luttikhuizen, P. C. , & Weissing, F. J. (2001). Sexual selection at the protein level drives the extraordinary divergence of sex‐related genes during sympatric speciation. Proceedings of the Royal Society B: Biological Sciences, 268, 2155–2161. 10.1098/rspb.2001.1780 PMC1088860 11600080

[jeb14120-bib-0108] van Doorn, G. S. , & Weissing, F. J. (2002). Ecological versus sexual selection models of sympatric speciation: A synthesis. Selection, 2, 17–40.

[jeb14120-bib-0109] Varea‐Sánchez, M. , Montoto, L. G. , Tourmente, M. , & Roldan, E. R. S. (2014). Postcopulatory sexual selection results in spermatozoa with more uniform head and flagellum sizes in rodents. PLoS One, 9, e108148. 25243923 10.1371/journal.pone.0108148 PMC4171531

[jeb14120-bib-0110] Vermeulen, A. , Engels, S. , Engqvist, L. , & Sauer, K. P. (2009). Phenotypic plasticity in sperm traits in scorpionflies (Mecoptera: Panorpidae): Consequences of larval history and seasonality on sperm length and sperm transfer. European Journal of Entomology, 106, 347–352.

[jeb14120-bib-0111] Vuarin, P. , Hingrat, Y. , Lesobre, L. , Jalme, M. S. , Lacroix, F. , & Sorci, G. (2019). Sperm competition accentuates selection on ejaculate attributes. Biology Letters, 15, 10–13. 10.1098/rsbl.2018.0889 PMC6451384 30890070

[jeb14120-bib-0112] Ward, P. I. (2000). Sperm length is heritable and sex‐linked in the yellow dung fly (*Scathophaga stercoraria*). Journal of Zoology, 251, 349–353.

[jeb14120-bib-0113] Weissing, F. J. , Edelaar, P. , & van Doorn, G. S. (2011). Adaptive speciation theory: A conceptual review. Behavioral Ecology and Sociobiology, 65, 461–480. 21423338 10.1007/s00265-010-1125-7 PMC3038232

[jeb14120-bib-0114] Westneat, D. F. , Sherman, P. W. , & Morton, M. L. (1990). The ecology and evolution of extra‐pair copulations in birds. Current Ornithology, 7, 331–369.

[jeb14120-bib-0115] Westneat, D. F. , & Stewart, I. R. K. (2003). Extra‐pair paternity in birds: Causes, correlates, and conflict. Annual Review of Ecology, Evolution, and Systematics, 34, 365–396.

[jeb14120-bib-0116] White, J. W. , Rassweiler, A. , Samhouri, J. F. , Stier, A. C. , & White, C. (2014). Ecologists should not use statistical significance tests to interpret simulation model results. Oikos, 123, 385–388.

[jeb14120-bib-0117] Wickham, H. , Averick, M. , Bryan, J. , Chang, W. , McGowan, L. D. , François, R. , Grolemund, G. , Hayes, A. , Henry, L. , Hester, J. , Kuhn, M. , Pedersen, T. L. , Miller, E. , Bache, S. M. , Müller, K. , Ooms, J. , Robinson, D. , Seidel, D. P. , Spinu, V. , … Yutani, H. (2019). Welcome to the tidyverse. Journal of Open Source Software, 4, 1686.

[jeb14120-bib-0118] Wilson, N. , Tubman, S. C. , Eady, P. E. , & Robertson, G. W. (1997). Female genotype affects male success in sperm competition. Proceedings of the Royal Society B: Biological Sciences, 264, 1491–1495.

