# Supplementary material for: Selection on sperm size in response to promiscuity and variation in female sperm storage organs
Source: J Evol Biol. 2022 Nov 10;36(1):131–43. doi: 10.1111/jeb.14120 (PMC10100110; doi:10.1111/jeb.14120)
Supplement: Supplementary file 1 — Appendix S1 [file JEB-36-131-s001.docx]

Supplementary information for “Selection on sperm size in response to promiscuity and variation in female sperm storage organs” Emily R. A. Cramer, Zelealem B. Yilma, Jan T. Lifjeld

*Reanalysis of the data of Hemmings et al. (2016)*

For each male separately, we used the package BEST (Kruschke & Meredith, 2021) to estimate the difference in sperm length between fecal samples (representing ejaculated sperm) and from the perivitelline layer (PVL) of his mate’s eggs. Data were downloaded from Dryad on 15 March 2022, https://doi.org/10.5061/dryad.vc7ss. Results are provided in Figure S1. Default settings were used, and default uninformative priors.


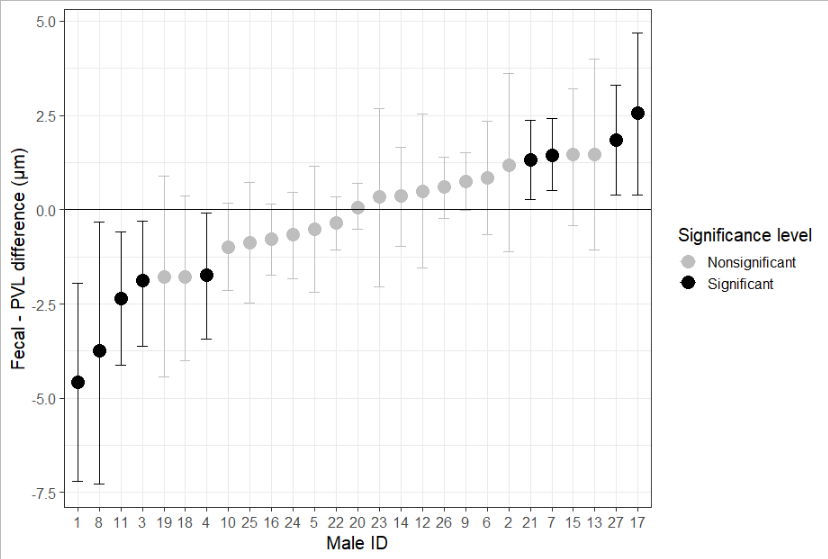


*Figure S1. Estimate and 95% Highest Density Interval from Bayesian comparison of sperm length on perivitelline layer and on in fecal sperm. Bird identity is the same as listed in Hemmings et al.’s data file. The males are sorted in order of the difference between fecal and PVL sperm (negative scores indicate sperm were shorter in the fecal sample). Points where the 95% HDI did not overlap 0 are in black; others are in grey.*

*Additional simulation conditions*

We ran additional simulation conditions to evaluate the impact of additional biological processes on our results. Except where noted otherwise, we maintain the population size of 200 males and 200 females, clutch size of 5 per female, and the normal distribution of sperm size with mean 0 and standard deviation 1. In the results shown below, simulations were run 100 times per set of conditions; pilot testing suggests that this is sufficient to give robust mean values, although the 95% quantile interval may be slightly less exact. We generally do not run the case where females copulate with only one male, to save computational time, but we run other combinations of promiscuity and among-female variation in storage site as in the main text. Generally, we also re-run the main conditions as a comparison point (resulting in slight variation in the quantile limits). For most simulation cases, we first present a figure comparable to Figure 3 in the main text, where each panel shows a different level of the newly introduced parameter; we then often present the same data rearranged in a second figure to more easily examine the impact of variation across levels of the new parameter. Most of these additional conditions are incorporated into the code uploaded to Zenodo.

*Social partners with repeated copulations*

For socially monogamous species with extra-pair paternity, it is widely assumed that the social partners copulate more often than extra-pair partners do. Empirical data on the relative copulation frequency of within-pair and extra-pair males is limited. However, Brommer *et al.* (2010) and Cramer *et al.* (2020) estimate a parameter, *s,* that reflects the relative probability that an extra-pair male fertilizes an egg, compared to a within-pair male. Across several species of passerine birds, Brommer *et al.* (2010) and Cramer *et al.* (2020) estimate *s* to range from 0.18 – 0.68. These results indicate that the within-pair male may be between 2 and 5 times more likely to fertilize each egg. Based on these data, we therefore simulate populations where the within-pair male copulates once (main model), twice or five times as often as the other (extra-pair) males. We add a higher level (ten times as many copulations by the within-pair male) to explore sensitivity to this parameter.

In the main model, we created a matrix of copulations for each population where each row was a female and each column contained a shuffled list of all males in the population. The number of columns was determined by the number of copulation partners for that simulation, and we ensured that no male appeared twice in any row. To simulate repeated copulations between social partners, we arbitrarily picked one column in the matrix and appended it to the matrix *a* additional times, where *a* is the number of additional copulations the within-pair male obtains (assuming all extra-pair males get one copulation). Note that this process allows each male to be the social partner to a single female.

Fertilizations were assigned probabilistically as described in the main text, i.e., using fit scores calculated using Eq. 1 as weights for R’s *sample* function. If the within-pair male has copulated multiple times, he now appears multiple times in the list of possible sires and therefore is more likely to be drawn. In effect, the within-pair male gets an (*a*+1) times advantage over an extra-pair male with the same fit score. Advantages associated with the fit scores, however, can be exponential, since Eq. 1 is an exponential equation. Stated another way, the within-pair male has more “raffle tickets” to the loaded raffle of sperm competition, while the loading of the raffle depends on relative sperm fit (Parker, 1990).

We find that the overall patterns of how selection responds to promiscuity and variation in the female sperm storage organs is very similar, regardless of the number of copulations a within-pair male obtains often (Figure S2).


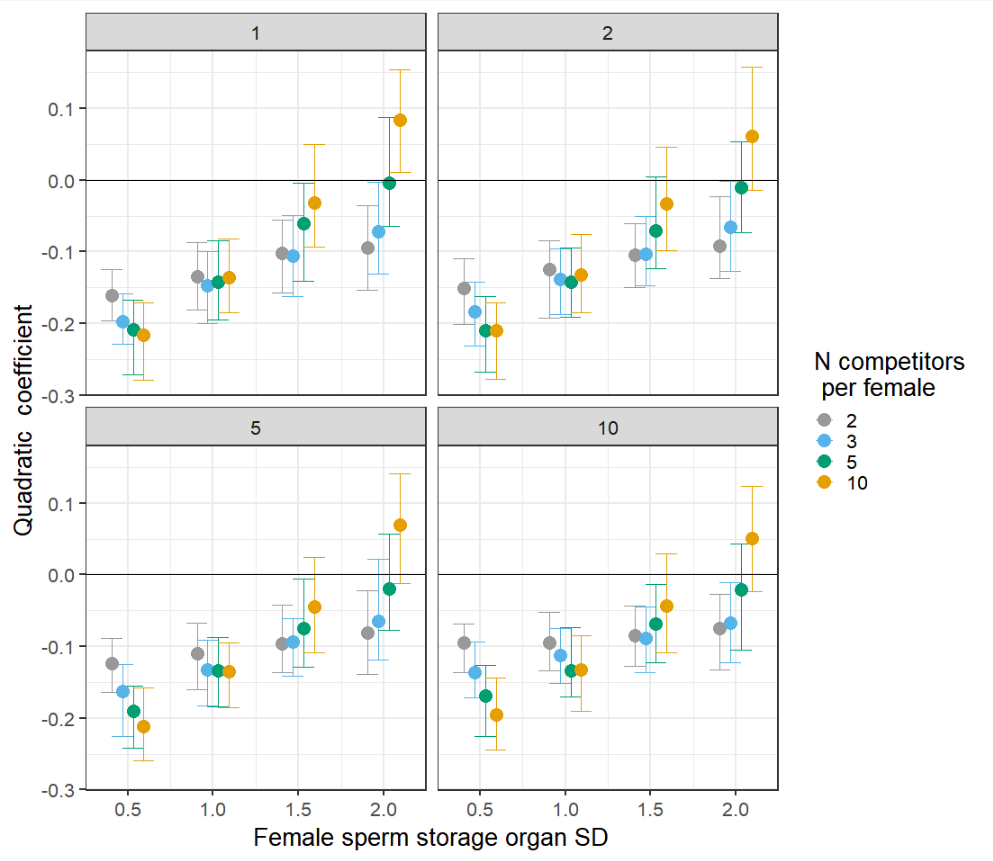
*Figure S2. The effect of repeated copulations between social partners on selection on sperm. Each panel shows a different number of copulations by the within-pair male, relative to single copulations by extra-pair males (i.e., all other males). Colors indicate promiscuity level. Points are medians, and bars show 95% quantiles.*

Where repeated copulations by the within-pair male affect selection estimates, they do so by reducing the strength of selection (Figure S3). This result is intuitive: the within-pair male is siring more offspring, regardless of how well his sperm fit the female, because he copulates more often, and this numerical advantage out-weighs the fit advantage. As a result, the impact of sperm morphology on reproductive success is reduced, and measured selection is weaker. However, the numerical advantage only rarely is of a similar order of magnitude to the fit advantage, since the former is multiplicative and the latter is exponential (Eq. 1).


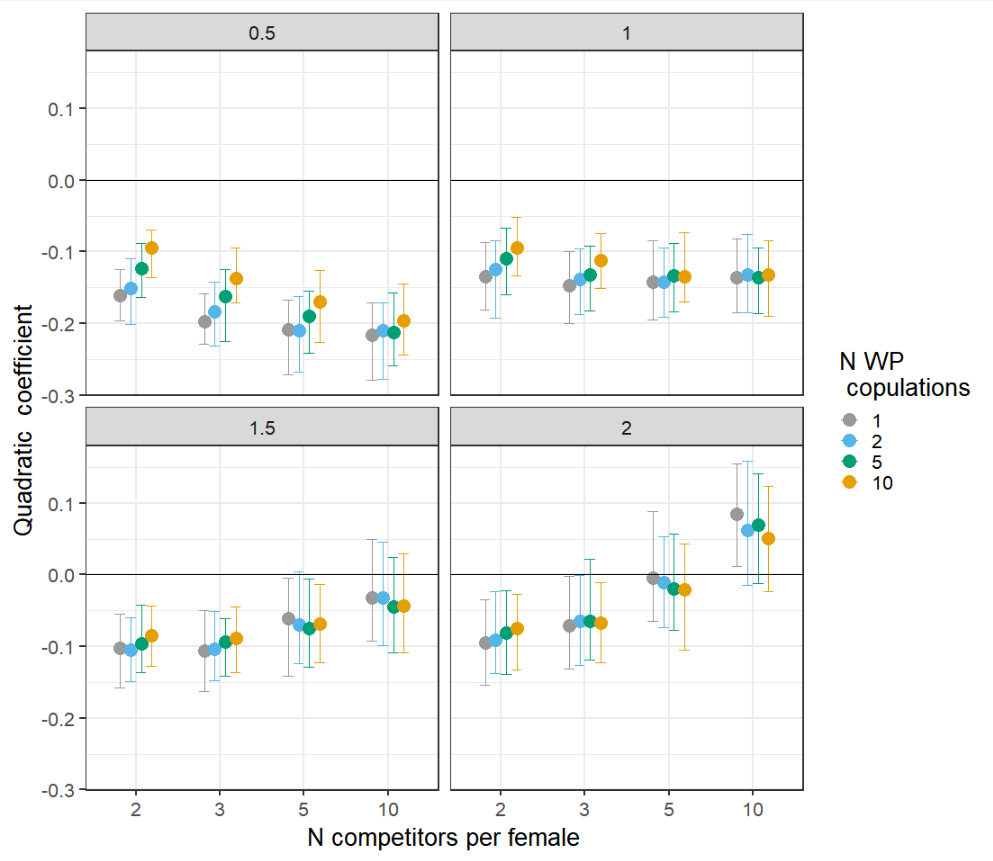
*Figure S3. The effect of repeated copulations between social partners on sperm selection dynamics. Each panel shows a different level of variation in female sperm storage organs. Colors indicate the number of copulations by the within-pair male, relative to single copulations by extra-pair males (i.e., all other males). Same data as Fig S2. Points are medians, and bars show 95% quantiles.*

Specifically, selection is impacted by repeated within-pair male copulations when both promiscuity and variation in female sperm storage organs are low (Figure S3, 2-5 competitors for SSO SD = 0.5, 2-3 competitors for SSO SD = 1 and 1.5, and for 2 competitors for SSO SD = 2). With high promiscuity, females are more likely to have copulated with at least one extra-pair male with an exceptionally good sperm fit (increasing the relative advantage due to fit), and the total number of copulations the female performs is higher (resulting in de-valuation of the repeated copulations of the within-pair male, relative to the total number of copulations). Therefore, the relative value of repeated copulations by the within-pair male is low.

With low promiscuity, females most likely copulate only with males with relatively moderate sperm size. If female sperm storage organ variation is low, these males are all relatively good fits for most females, so that variation in the fit scores among males is of a similar order of magnitude to the multiplicative advantage due to repeated copulations, allowing the repeated copulations by the within-pair male to substantially alter selection. If female sperm storage organ variation is high, males with moderate sperm size may be relatively poor fits, and variation in the fit scores may be more extreme (i.e., of a substantially greater magnitude than multiplicative differences), so that repeated copulations by the within-pair male do not have a strong impact on selection.

*Threshold model: extreme mismatches cause sperm to not be stored*

It is plausible that some sperm may fit female sperm storage organs too poorly to achieve be stored, and thus to achieve fertilization. To explore how this possibility would impact our results, we introduced a threshold in the calculation of sperm-sperm storage organ fit, beyond which all sperm were given a fit of 0, and therefore were not stored and could not fertilize eggs. Females that only copulated with males with sperm outside this threshold therefore produced no fertilized eggs. We assumed that this threshold was symmetrical for exceptionally short or long sperm: specifically, if the absolute value of the difference between sperm and sperm storage organ was greater than the threshold, the fit score was 0, while in other cases it was calculated by Eq. 1.

We somewhat arbitrarily chose threshold values of 1, 2, 4, and 6 size units (recall that the standard deviation for sperm size was always set to 1, so about 95% of males are within 4 size units of each other and over 99% of males are within 6 size units of each other). The lower values seem unlikely to be biologically relevant, as they would imply that a large number of conspecific males are unable to fertilize a female’s eggs, but we *post hoc* decided to include them as a logical proof-of-concept that the threshold can impact selection. Here we also included the case where females copulate with only a single male, expecting that stabilizing selection would result.

We find that the overall patterns of how selection responds to promiscuity and variation in the female sperm storage organs is generally similar (Figure S4), except that there can be stabilizing selection on sperm also when females copulate with only one male (i.e., when there is no sperm competition but only cryptic female choice). With a low threshold (1 size unit), stronger stabilizing selection occurs when the among-female variation is low, and stronger disruptive selection when the among-female variation is high (Figure S5). This is intuitive, as we have essentially narrowed the within-female preference function, making an excellent fit between sperm and sperm storage organ necessary for fertilization. However, we emphasize that this condition seems unlikely to be relevant in most circumstances, as it implies that a large number of conspecific males are unable to fertilize individual females. Selection was highly similar when the threshold was greater than or equal to 2 and females mated multiply (Figures S5). Fit scores for sperm-sperm storage organs that are more than 2 units different (calculated via Eq. 1) are substantially low, so that replacing them with a value of 0 has minimal impact on overall selection.


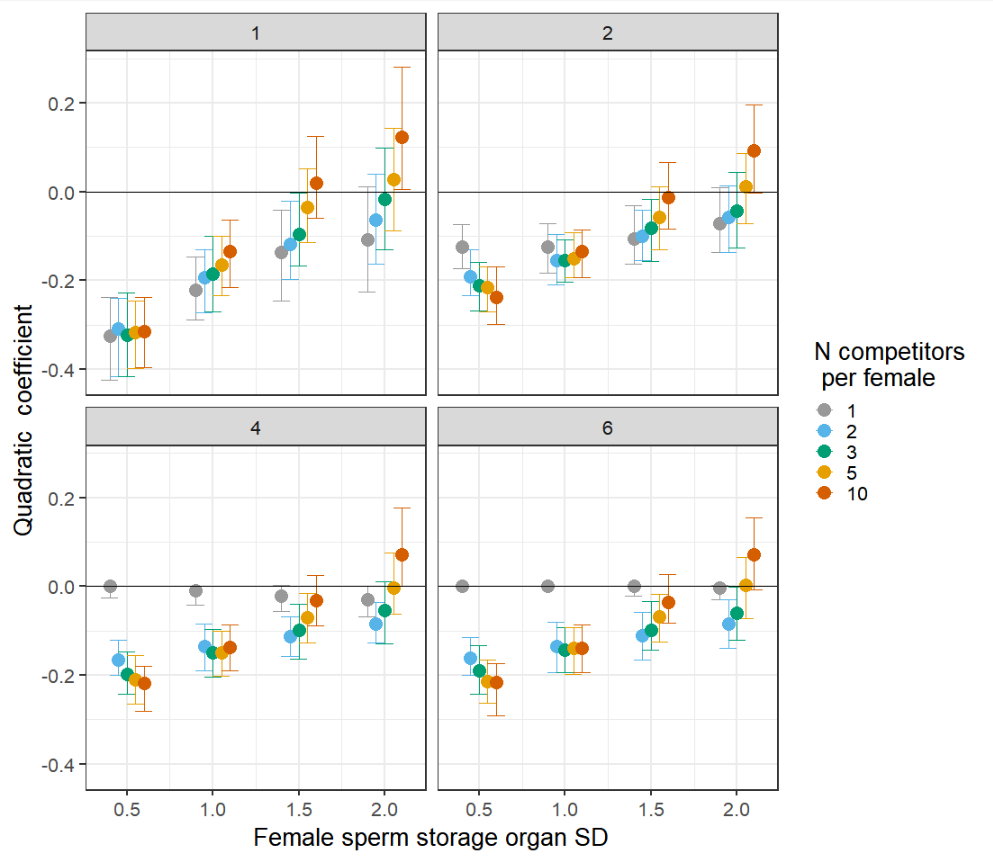
*Figure S4. The effect of a threshold for storage success on sperm selection dynamics. Each panel shows a different value of the threshold distance between sperm and female sperm storage organ, beyond which sperm fail to be stored. Colors indicate promiscuity level. Points are medians, and bars show 95% quantiles.*


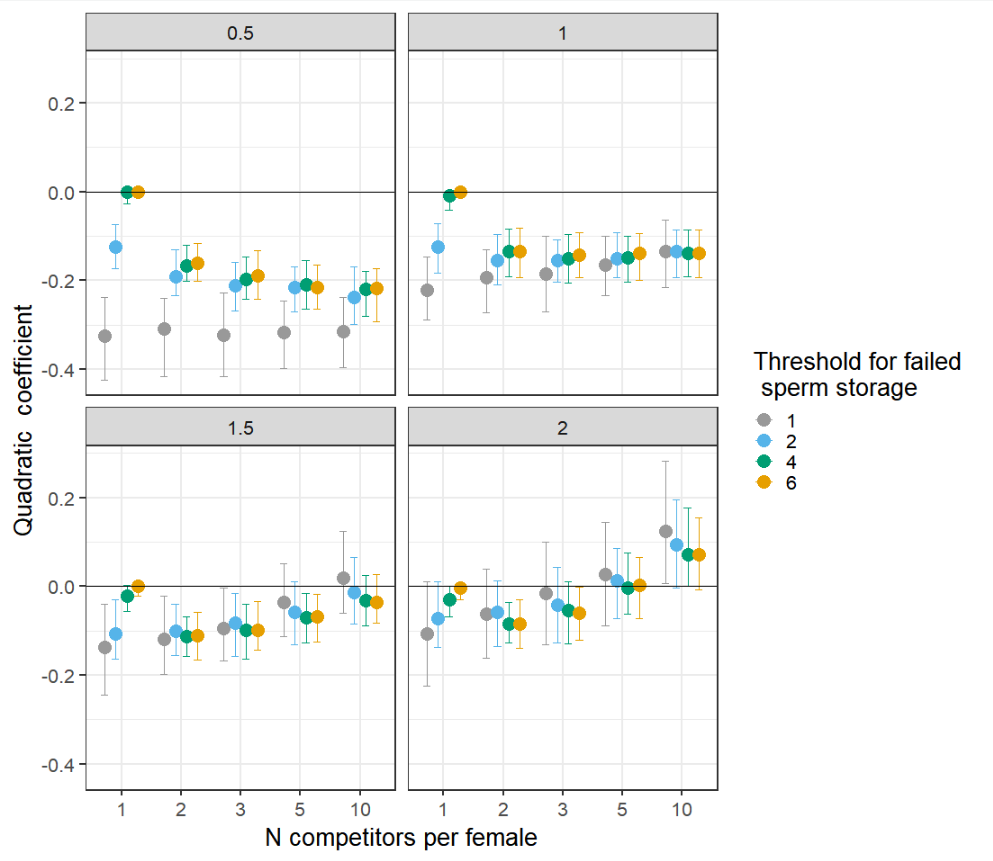


*Figure S5. The effect of a threshold for fertilization success on sperm selection dynamics. Each panel shows a different level of variation in female sperm storage organs. Colors indicate the absolute value of the difference between sperm and sperm storage organ size, beyond which sperm fail to be stored. Same data as Fig S4. Points are medians, and bars show 95% quantiles.*

Notably, some females copulated with no males whose sperm was a good enough fit to fertilize eggs. This occurred more often with lower promiscuity (i.e., fewer males sampled, so less opportunity to sample a good male) and when variation in female sperm storage organs was greater (leading to some females being relatively poor fits for most males, Figure S6).


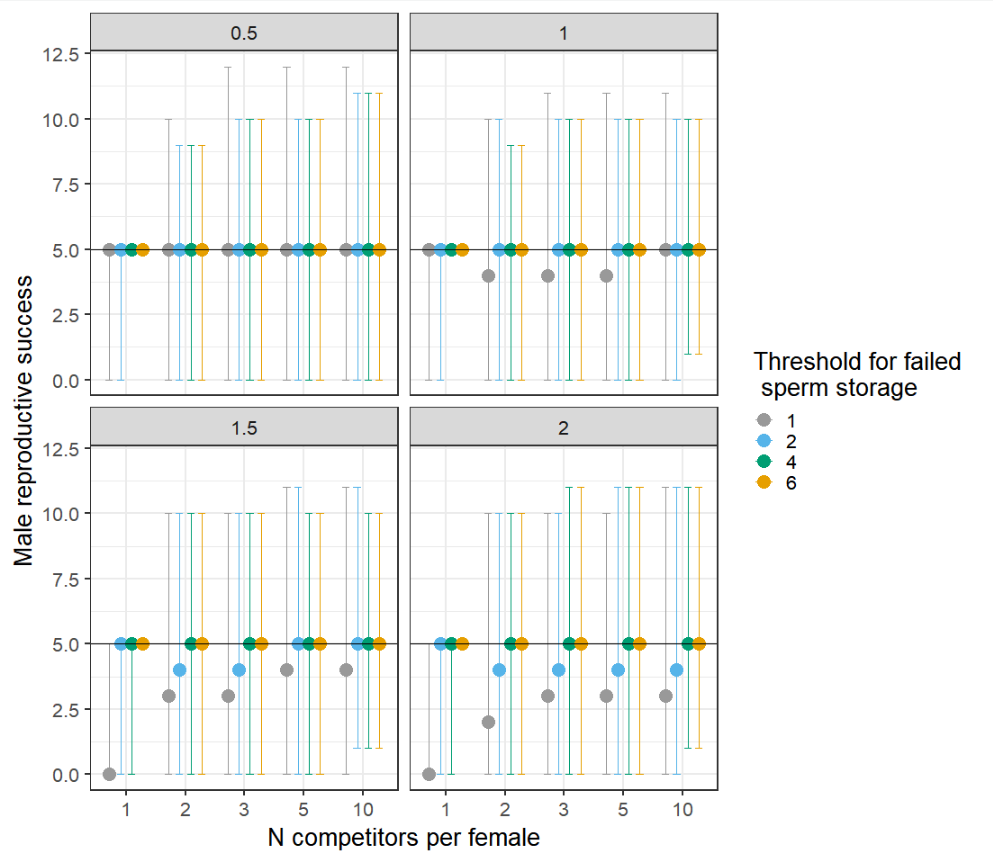
*Figure S6. The effect of a threshold for storage success on mean male reproductive success. Each panel shows a different level of variation in female sperm storage organs. Colors indicate the absolute value of the difference between sperm and sperm storage organ size, beyond which sperm fail to be stored. Since population mean male and female reproductive success by definition are equal, deviations from the value of 5 (i.e., clutch size) indicate instances where not all eggs were fertilized. In turn, this implies that some females did not copulate with any males whose sperm was within the acceptable range. Points are medians, and bars show 95% quantiles.*

*Directional selection. Adding directional selection*

In several taxa, longer sperm are more likely to fertilize eggs (for example, (Miller & Pitnick, 2002; Bennison *et al.*, 2015). One mechanism by which this pattern could, in principle, arise, is if female sperm storage organs become longer than sperm, due to drift or other processes. We therefore explore populations where the mean female sperm storage organ is longer than the mean sperm. We arbitrarily chose to increase the mean for females by 0.25, 0.5, and 1 size units. As in the main simulation, the distribution of sperm size has a standard deviation of 1 and a mean of 0.

This offset was effective in causing directional selection (Figure S7). The overall patterns of how quadratic selection responds to promiscuity and variation in the female sperm storage organs is generally similar across levels of imposed directional selection (Figure S8), although quadratic coefficients generally have higher values as directional selection increases (Figure S9). That is, stabilizing selection is weaker, and disruptive selection stronger, with a greater difference between mean values for sperm storage organs and sperm. Stabilizing selection becomes weaker as the male-female difference increases, because males with mean phenotypes are less well-fit to the majority of females. The quadratic coefficient no longer shows symmetrical disruptive selection (see figure 2 in the main text), but instead an asymmetrical curve (Figure S10).


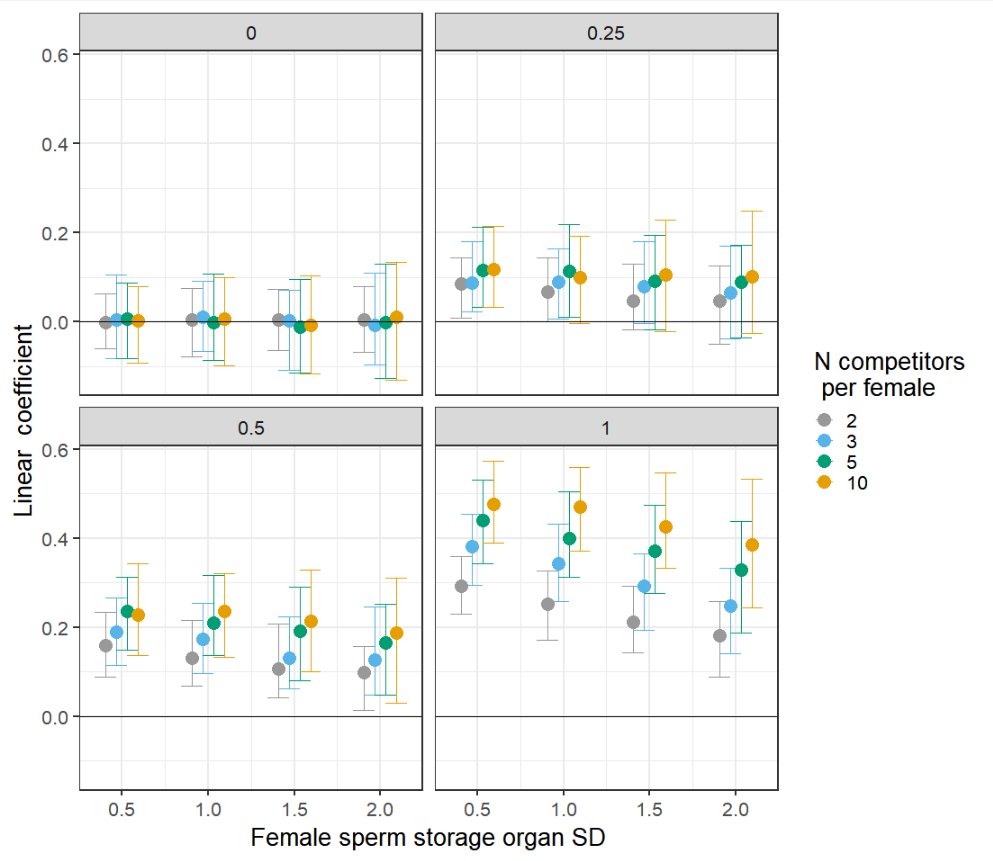
*Figure S7. Verification that directional selection (the linear selection coefficient) increased with a larger difference in the population mean values for female sperm storage organs and sperm (panels). Directional selection was also stronger with higher promiscuity (colors). Points are medians, and bars show 95% quantiles.*


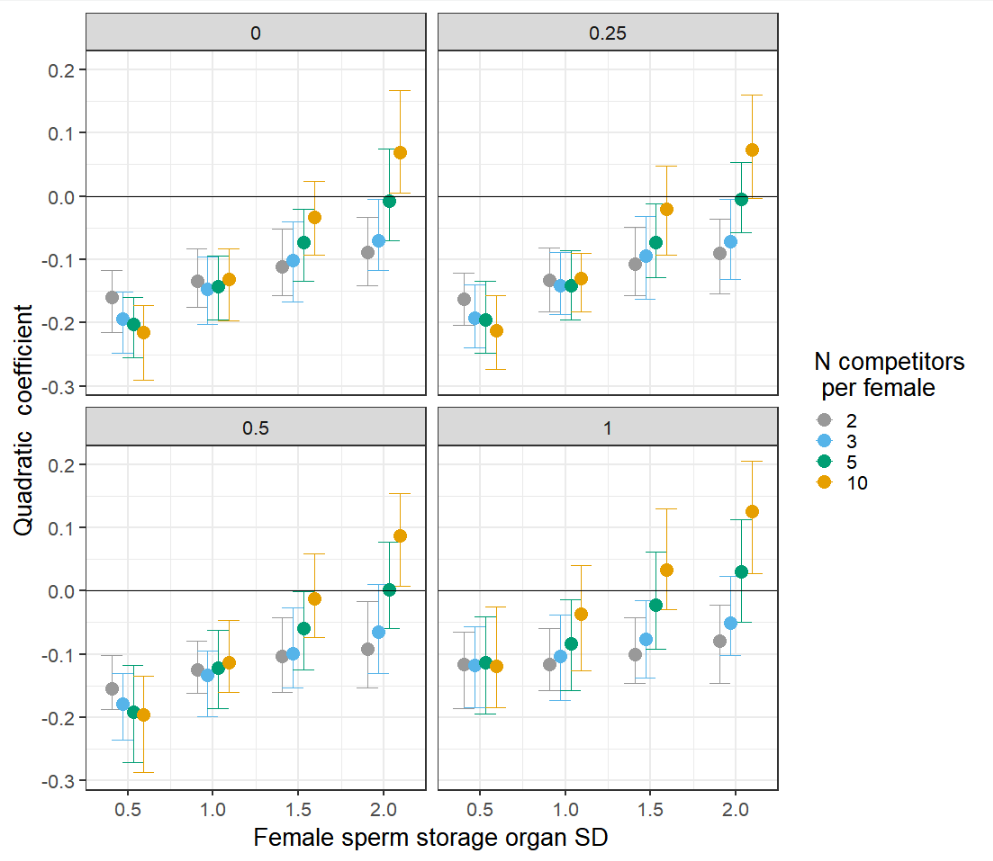
*Figure S8. Quadratic selection coefficients on sperm size in populations with directional selection. Each panel shows a different value for the difference between mean female sperm storage organ size and mean sperm size of the population. Colors are promiscuity levels. Points are medians, and bars show 95% quantiles.*


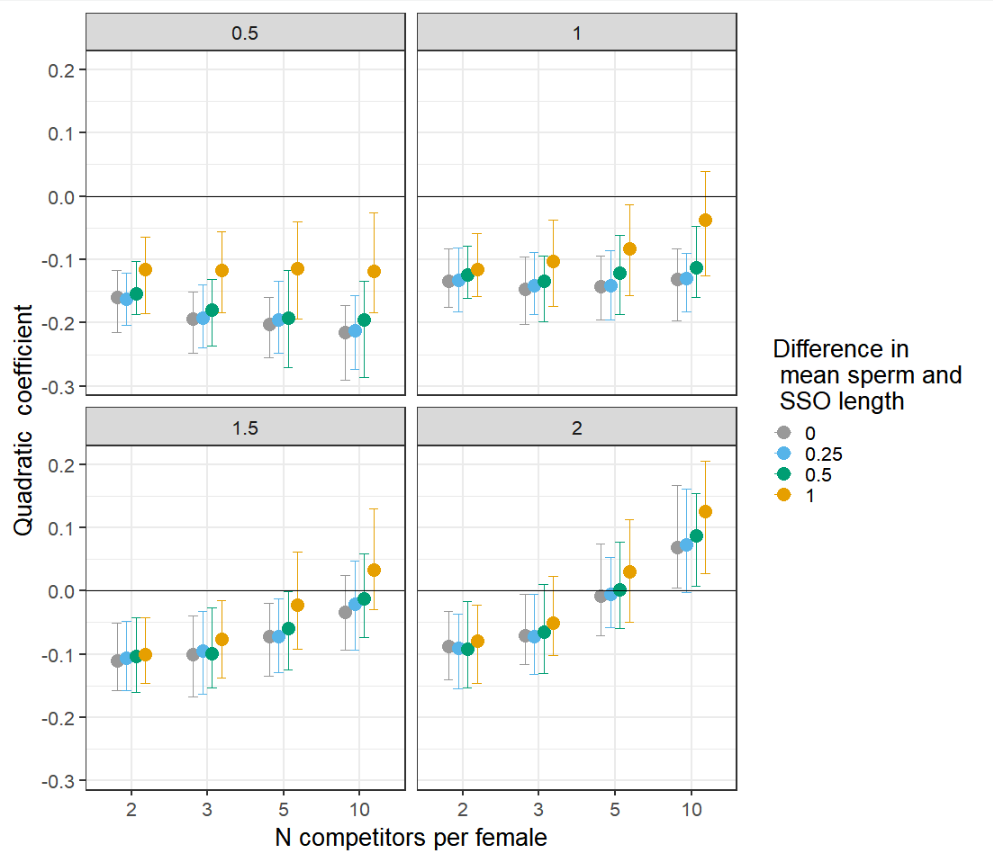
*Figure S9. Quadratic selection coefficients on sperm size in populations with directional selection. Each panel shows a different level of variation in female sperm storage organs. Colors indicate the difference between mean female sperm storage organ size and mean sperm size of the population. Same data as Fig. S8. Points are medians, and bars show 95% quantiles.*


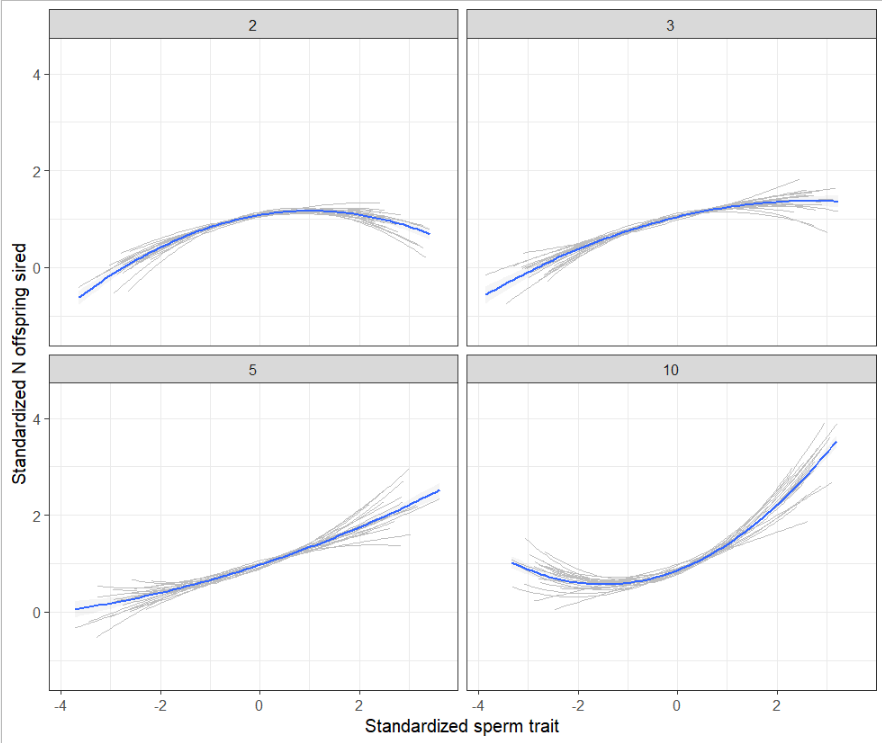
*Figure S10. Example selection gradients from populations where the difference in mean sperm and sperm storage organ size is one and the standard deviation in female sperm storage organs is two, with each panel showing a different number of competing males. Grey lines are individual populations, relating the reproductive success of individual males to their sperm sizes. The blue line is the overall mean for the populations included in the plot.*

As an alternate approach to produce directional selection, we assumed that sperm that longer than the female sperm storage organ might be stored less successfully than sperm that are shorter than the female sperm storage organ. To simulate this circumstance, we created an asymmetrical preference function, so that the spread of the function was reduced for longer sperm, compared to shorter sperm. To achieve this, we used the fit scores produced by Eq. 1 for sperm that were of equal size or smaller than the female’s sperm storage organ. For larger sperm, we modified Eq. 1 by dividing $\sigma_{U}$ by 2 (an arbitrarily chosen value).

Because this approach narrowed the total female preference function, selection was overall more disruptive (Figure S11), as expected according to the analytical model results. However, overall patterns were similar to those from the main model. Directional selection toward shorter sperm was also found under the asymmetric model (data not shown).


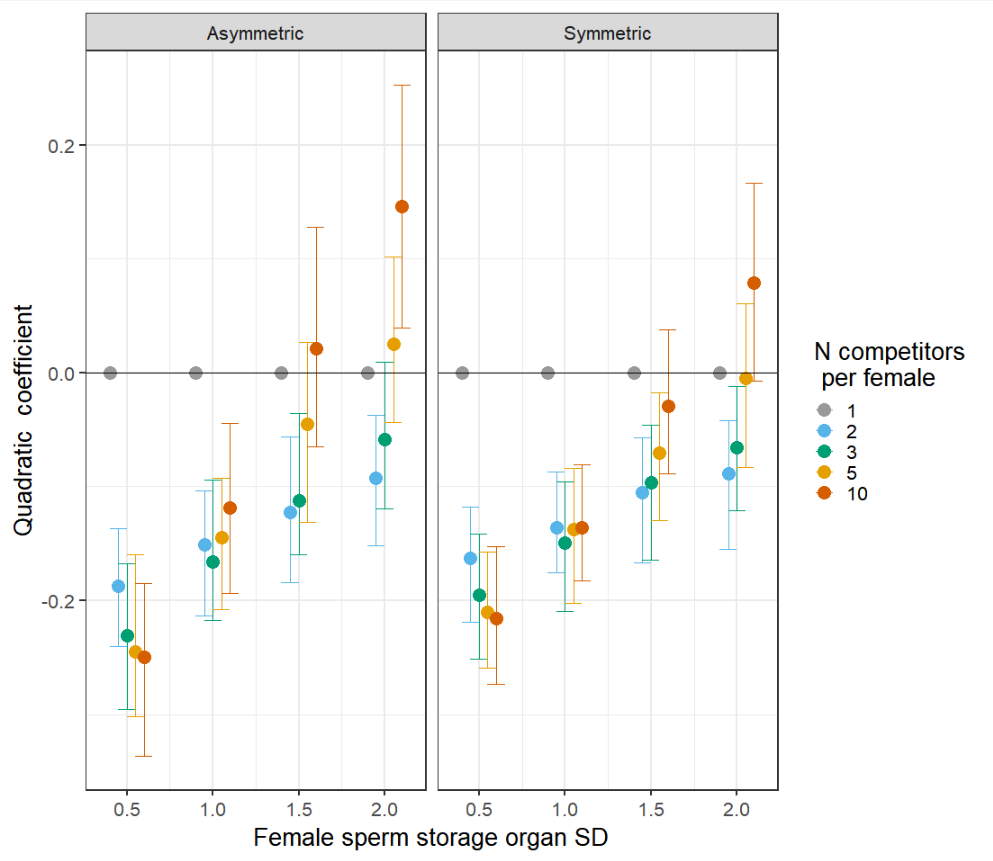
*Figure S11. The effect of asymmetric preference for longer versus shorter sperm on quadratic selection on sperm. Panels show the symmetric case (as in the main model) or the asymmetric case, where there is stronger selection against sperm that are longer than the sperm storage organs than against sperm that are shorter than the sperm storage organs. Colors indicate promiscuity level. Points are medians, and bars show 95% quantiles.*

*Within-female variation:*

Within an individual female, there may be multiple sperm storage organs with different sizes (Briskie, 1996), and sperm from different males can be stored in separate organs from each other (Hemmings & Birkhead, 2017). We sought to explore how this variation would impact our main patterns. However, we expect substantial variation among taxa in how intra-individual sperm storage organ size is distributed, and how (or if) certain sperm storage organs within each female are more likely to support the sperm that will be used for fertilization. To minimize added model complexity, we therefore use the breadth of the preference function as a proxy for the likely effect of intra-individual variation in sperm storage organs. To do so, we simply changed the value of $\sigma_{U}$ in Eq. 1. We arbitrarily chose values of 0.5, 1, 1.5, and 2.

Increasing the breadth of the preference function generally dampens the strength of quadratic selection, converging towards weak stabilizing selection (for all levels of variation in female sperm storage organs and number of competing males) with increasing breadth (Figure S12). With sufficiently broad preference functions, disruptive selection does not occur even with high among-individual variation in female sperm storage organs, because each individual female is less selective for well-matched sperm. The idea that broader individual female preference functions should be less likely to lead to disruptive selection has been thoroughly explored, for example in the sympatric speciation literature (e.g., Weissing *et al.*, 2011).


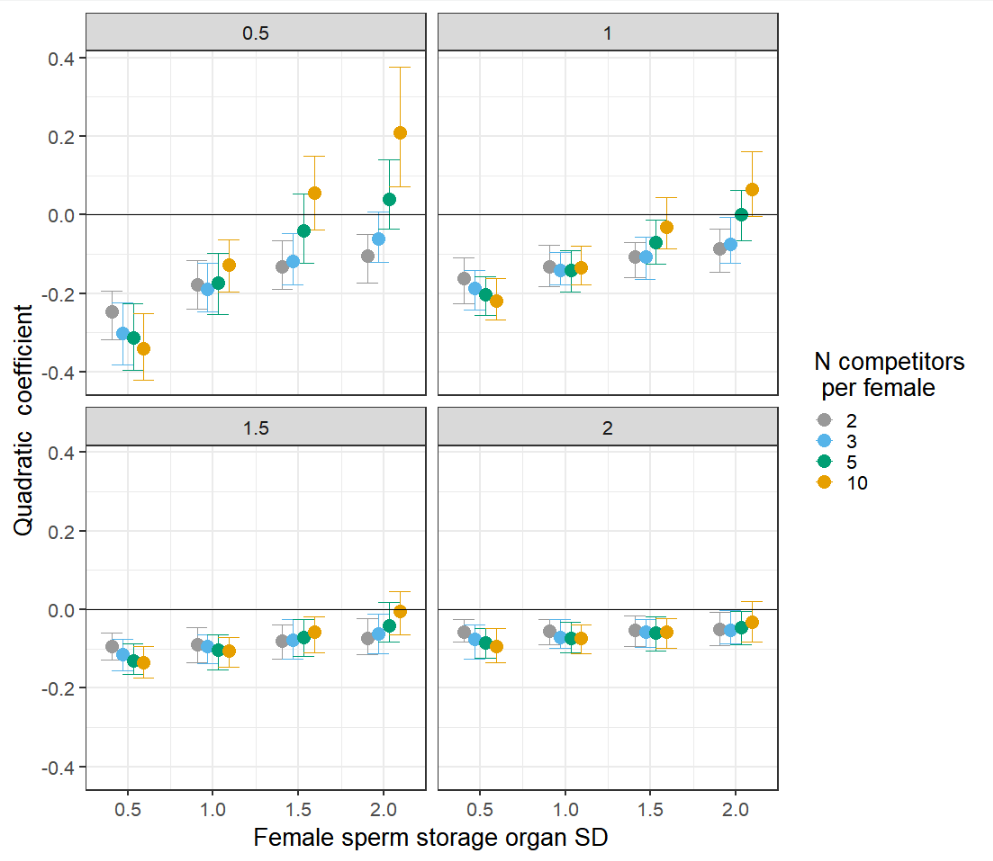
*Figure S12. Quadratic selection coefficients on sperm size. Each panel shows a different value for*  $\sigma_{U}$ *(i.e., breadth of the preference function, Eq. 1). Colors indicate promiscuity level. Points are medians, and bars show 95% quantiles.*

We expect that these results are representative of how intra-individual variation in sperm storage tubule size would impact selection on sperm, as long as intra-individual variation was unimodally and symmetyrically distributed, and no fertilization advantage was associated with a particular subset of sperm storage organs. Some element of directional selection could be expected if the intra-individual distribution of sperm storage organs were skewed and/or if certain sperm storage organ size within females provided a fertilization advantage. However, given the results of the simulations where we introduced directional selection (previous section), we do not expect that our main model results would be strongly affected.

*Shared paternity*

Many mate choice models do not have shared paternity, since they typically allow a female to copulate with only one male. We evaluated whether sharing paternity impacts selection by altering the simulation so that the single copulation partner with the best-fit sperm fertilized all 5 eggs.

We found that sharing paternity weakened selection strength relative to iterations where the best-fit sperm fertilized all eggs (Figure S13). However, the main patterns are robust, with selection being either stabilizing or disruptive with high promiscuity, depending on the level of variation in the female preference. When females copulated with only fewer males, selection was generally stabilizing.


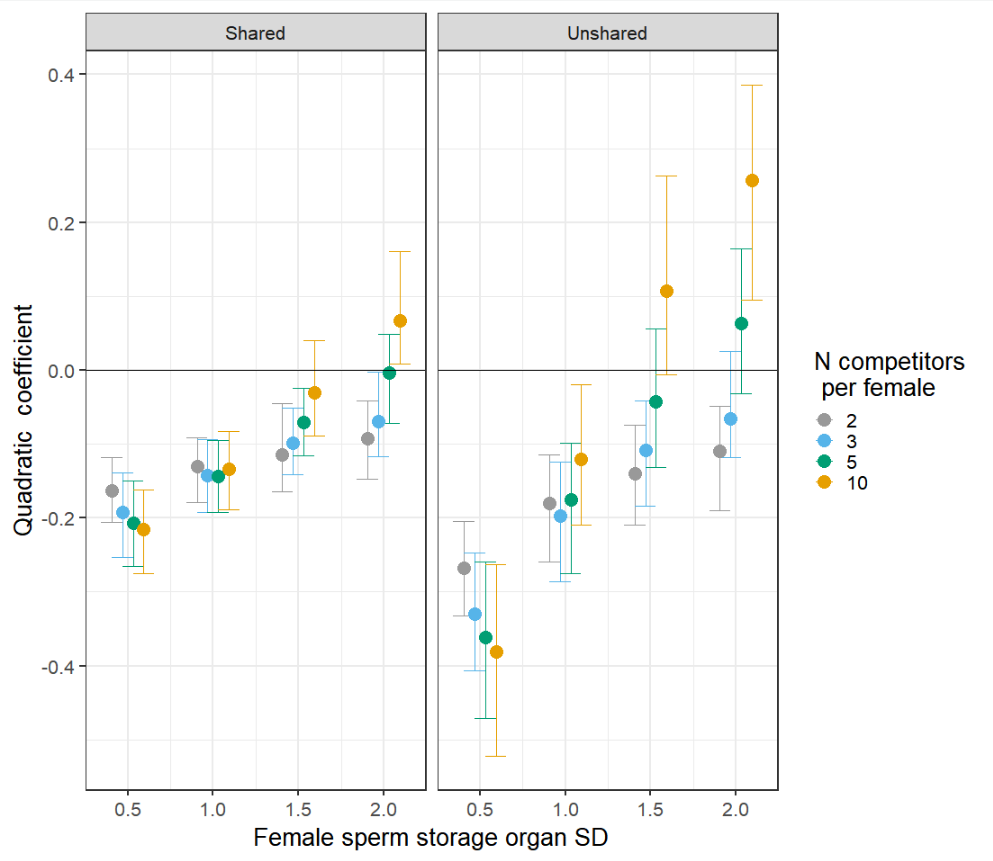
Figure S13. *Quadratic selection coefficients on sperm size, where paternity was either shared among males depending on relative fit to the sperm-sperm storage organ (“shared”; main results) or where all paternity was assigned to the single copulation partner with the best-fitting sperm (“unshared”). Colors indicate promiscuity level. Points are medians, and bars show 95% quantiles.*

*Male and female promiscuity separately*

Finally, we wanted to evaluate the impact of male and female promiscuity separately. In the main simulations, for efficiency and due to the population being a closed population, we coded the simulations such that all individuals of both sexes had the same number of copulation partners. Here, we instead generated populations of 200,000 males and 200,000 females as described in the main text. We *a priori* chose 200 males to be focal subjects, and for those 200 males only, we drew 2, 5, or 10 female copulation partners (at random, without replacement). Each of these females in turn drew 2, 5, or 10 copulation partners from the remaining male pool (without replacement). Thus there were no repeated copulations between pairs of individuals within the dataset, and the number of male and female copulation partners was altered independently. The same females may have been drawn by different focal males, but this was treated as an independent breeding attempt for that female (i.e., she drew another set of copulation partners for that breeding attempt). All paternity by focal males was included (both when he was the focal male and if he was selected as a competitor, though that appears unlikely). We note that this is a somewhat incomplete picture, as we did not simulate breeding for 199,800 males, where the focal males could have also been competitors. However, we expect that to add noise rather than bias to the results. Because this simulation approach was more computationally demanding, we used only a subset of conditions evaluated in the main paper (2, 5, and 10 partners, with female preference SD of 0.5, 1.5, and 2).

Variation in the number of females a focal male copulated with did not impact mean parameter estimates, though it reduced the variance (Figure S14). Female promiscuity strongly affected selection on sperm, matching the effects of number of copulation partners in the main manuscript. We therefore conclude that female promiscuity, rather than male promiscuity, drives the patterns in the main text.


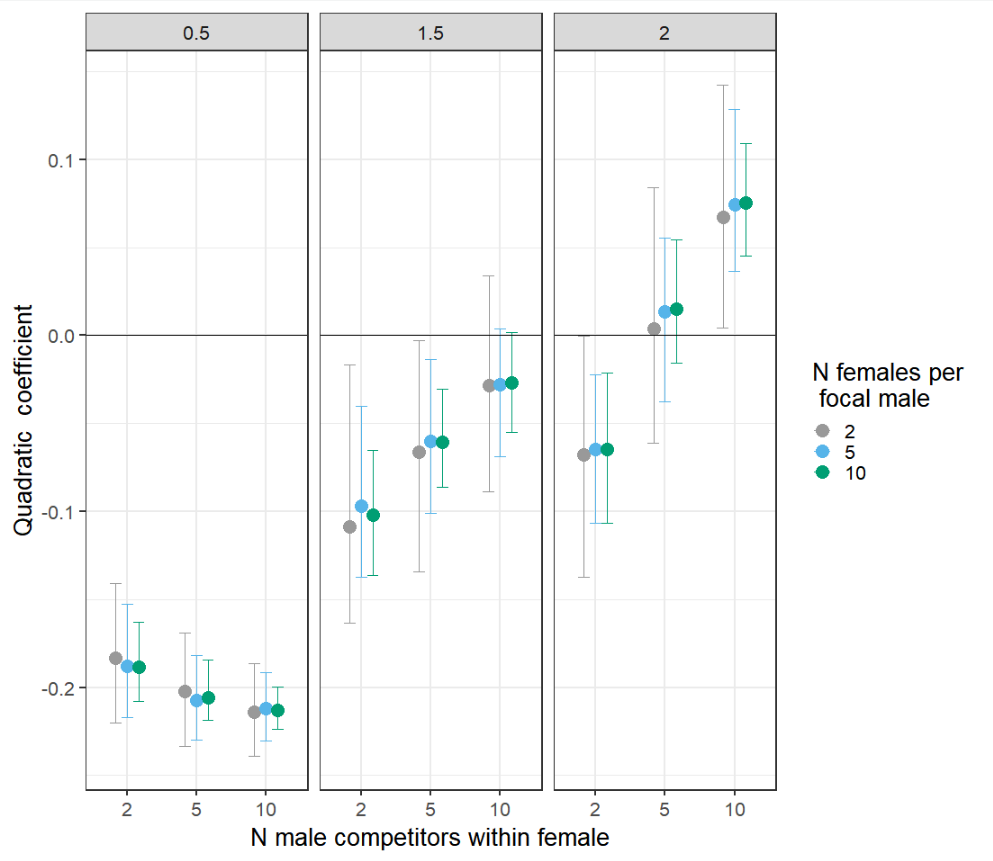


*Figure S14. Effects of independently varying the number of females each focal male copulates with (colors) and the number of males each female copulates with (i.e., number of sperm competitors for a particular set of eggs; x axis).Results shown for three levels of among-female variation in sperm storage organ size (panels). Points are medians, and bars show 95% quantiles.*

*Verifying analytical model predictions*

The analytical model predicts no quadratic selection when where ${\sigma_{F}}^{2}={\sigma_{M}}^{2}+{\sigma_{U}}^{2}$(where ${\sigma_{F}}^{2}$is the variance for the whole population in female preference; ${\sigma_{M}}^{2}$ is the variance in sperm size; and ${\sigma_{U}}^{2}$is the variance in the preference function for each female, which we did not vary in the main simulations). We arbitrarily chose three sets of values for these three terms that satisfy the equation given above, and ran 100 iterations of the simulation for each set of conditions. Here we allowed each individual to copulate once with each member of the opposite sex (i.e., 200 copulations).

As predicted by the analytical model, quadratic selection was 0 for these conditions (Table S1).

| Table S1. Median and 95% quantiles for simulations where the expected value of the quadratic selection coefficient is 0, based on the analytical model (based on 100 iterations). | | | |
| --- | --- | --- | --- |
| ${\sigma_{F}}^{2}$ | ${\sigma_{M}}^{2}$ | ${\sigma_{U}}^{2}$ | Quadatic selection coefficient |
| 2 | 1 | 1 | 0.000 (-0.063, 0.051) |
| 4 | 2 | 2 | 0.003 (-0.059, 0.063) |
| 7 | 4 | 3 | -0.000 (-0.086, 0.073) |

*Literature cited*

Bennison, C., Hemmings, N., Slate, J. & Birkhead, T. 2015. Long sperm fertilize more eggs in a bird. *Proc. R. Soc. B Biol. Sci.* **282**: 20141897.

Briskie, J. V. 1996. Spatiotemporal of sperm storage and last-male patterns in birds sperm precedence. *Funct. Ecol.* **10**: 375–383.

Brommer, J.E., Alho, J.S., Biard, C., Chapman, J.R., Charmantier, A., Dreiss, A., *et al.* 2010. Passerine extrapair mating dynamics: A Bayesian modeling approach comparing four species. *Am. Nat.* **176**: 178–187.

Cramer, E.R.A., Kaiser, S.A., Webster, M.S. & Ryder, T.B. 2020. Common field data limitations can substantially bias sexual selection metrics. *Am. Nat.* **196**: 180–196.

Hemmings, N. & Birkhead, T. 2017. Differential sperm storage by female zebra finches *Taeniopygia guttata*. *Proc. R. Soc. B Biol. Sci.* **284**.

Kruschke, J. & Meredith, M. 2021. BEST: Bayesian Estimation Supersedes the t-Test. R package version 0.5.4.

Miller, G.T. & Pitnick, S. 2002. Sperm-female coevolution in *Drosophila*. *Science*  **298**: 1230–1233.

Parker, G.A. 1990. Sperm competition games: raffles and roles. *Proc. R. Soc. London B* **242**: 120–126.

Weissing, F.J., Edelaar, P. & van Doorn, G.S. 2011. Adaptive speciation theory: A conceptual review. *Behav. Ecol. Sociobiol.* **65**: 461–480.
